# Power Efficiency Enhancement of Organic Light-Emitting Diodes Due to the Favorable Horizontal Orientation of a Naphthyridine-Based Thermally Activated Delayed Fluorescence Luminophore

**DOI:** 10.1021/acsaelm.2c01529

**Published:** 2023-01-25

**Authors:** Rasa Keruckiene, Eimantas Vijaikis, Chia-Hsun Chen, Bo-Yen Lin, Jing-Xiang Huang, Chun-Chieh Chu, Yi-Chung Dzeng, Chi Chen, Jiun-Haw Lee, Tien-Lung Chiu, Simas Macionis, Jonas Keruckas, Rita Butkute, Juozas Vidas Grazulevicius

**Affiliations:** † Department of Polymer Chemistry and Technology, Kaunas University of Technology, K. Barsausko St. 59, LT-50254Kaunas, Lithuania; ‡ Materials Science and Engineering and Physics, Graduate Institute of Photonics and Optoelectronics and Department of Electrical Engineering, National Taiwan University, Taipei10617, Taiwan; § Department of Opto-Electronics Engineering, National Dong Hwa University, Shoufeng, Hualien974301, Taiwan; ∥ Research Center for Applied Sciences, Academia Sinica, Taipei11529, Taiwan; ⊥ Department of Electrical Engineering, Yuan Ze University, Chung-Li32003, Taiwan

**Keywords:** naphthyridine, angle-dependent photoluminescence, grazing-incidence small-angle X-ray scattering, thermally activated delayed fluorescence, molecular orientation, OLED

## Abstract

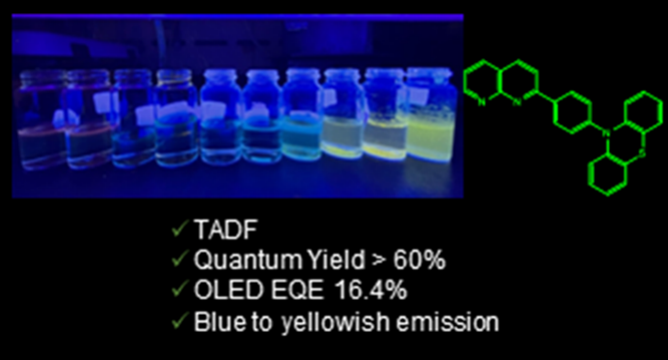

Four emitters based on the naphthyridine acceptor moiety and various donor units exhibiting thermally activated delayed fluorescence (TADF) were designed and synthesized. The emitters exhibited excellent TADF properties with a small Δ*E*_ST_ and a high photoluminescence quantum yield. A green TADF organic light-emitting diode based on 10-(4-(1,8-naphthyridin-2-yl)phenyl)-10*H*-phenothiazine exhibited a maximum external quantum efficiency of 16.4% with Commission Internationale de L’éclairage coordinates of (0.368, 0.569) as well as a high current and power efficiency of 58.6 cd/A and 57.1 lm/W, respectively. The supreme power efficiency is a record-high value among the reported values of devices with naphthyridine-based emitters. This results from its high photoluminescence quantum yield, efficient TADF, and horizontal molecular orientation. The molecular orientations of the films of the host and the host doped with the naphthyridine emitter were explored by angle-dependent photoluminescence and grazing-incidence small-angle X-ray scattering (GIWAXS). The orientation order parameters (Θ_ADPL_) were found to be 0.37, 0.45, 0.62, and 0.74 for the naphthyridine dopants with dimethylacridan, carbazole, phenoxazine, and phenothiazine donor moieties, respectively. These results were also proven by GIWAXS measurement. The derivative of naphthyridine and phenothiazine was shown to be more flexible to align with the host and to show the favorable horizontal molecular orientation and crystalline domain size, benefiting the outcoupling efficiency and contributing to the device efficiency.

## 1 Introduction

Organic light-emitting diodes (OLEDs) have made great progress in display technologies due to the advantages of low weight, low power consumption, and high color saturation.^1^ Organic compounds exhibiting phosphorescence (Ph) and thermally activated delayed fluorescence (TADF) are versatile candidates for the improvement of device efficiency by extracting nonradiative triplet excitons due to their ability of strong spin orbital coupling (SOC) and small singlet–triplet splitting (Δ*E*_ST_).^2−4^ The internal quantum efficiency (IQE) of OLEDs based on these systems can reach 100%, which is considerably higher than that of the devices based on conventional fluorescent materials (25%). However, to enhance the efficiency of intersystem crossing (ISC), phosphorescent emitters require the incorporation of heavy metal atoms,^5^ which can form as trap centers during charge injection and recombination. Meanwhile, for efficient TADF, the design of donor–acceptor molecular architectures with strong steric hindrance is a prerequisite.^2^ Thus, the distribution of the highest occupied molecular orbital (HOMO) and the lowest unoccupied molecular orbital (LUMO) can be separated, and the intramolecular charge transfer state can be induced, resulting in small Δ*E*_ST_.^6,7^

Easily obtainable and efficient materials are preferred for the advancement of OLED technologies. The naphthyridine moiety has recently attracted attention as the acceptor moiety of the design of donor–acceptor (D–A) electroactive compounds.^8−10^ The cyclization of amino-substituted phenylaldehyde can be performed using green chemistry methods that allow a mild and greener synthesis of substituted naphthyridines.^11^ They have attracted interest due to their electron-deficient characteristic, tunable luminescence, and ease of structural modification and processing.^12^ D–A systems, in which the naphthyridine moiety was used as an electron acceptor, were successfully used in host–guest systems of OLEDs.^8−10^ A series of bipolar host materials, composed of an electron-transporting naphthyridine moiety and a phenylene bridge with hole-transporting carbazole or diphenylamine, were reported.^10^ The highest external quantum efficiency (EQE) of OLEDs with the green TADF emitter 1,2,3,5-tetrakis(carbazol-9-yl)-4,6-dicyanobenzene (4CzIPN) was 18.4%, with power efficiency (PE) values reaching 53.8 lm/W.^10^ Derivatives of naphthyridine, in which the dimethylacridan moiety was used as an electron donor, were found to be efficient TADF emitters. The external efficiencies of OLEDs prepared using such emitters reached 14.1% with a PE value of 36.9 lm/W^13^ and 16.8% with a PE value of 50.7 lm/W.^8^ The devices exhibited relatively low turn-on voltages and low efficiency roll-off because of the twisted conformation and the aggregation-induced delayed fluorescence (AIDF) of the emitters. Naphthyridine-based D–A-type TADF emitters, in which carbazole was used as the donor moiety, were found to be even more efficient.^14,15^ The deep blue OLED reached an EQE of 17.6%,^14^ and the EQE of the green OLED was up to 20.9%, with a PE value of 26.5 lm/W.^15^ The other electron-rich moieties that are commonly used in TADF compounds, such as phenothiazine^9^ or phenoxazine,^16−18^ have not yet been extensively investigated in naphthyridine-based D–A systems. OLEDs based on TADF emitters containing 1,5-naphthyridine as an electron acceptor moiety and phenoxazine or phenothiazine as electron donor units exhibited high external quantum efficiencies of 29.9 and 25.8% and maximum luminance values of 33,540 and 14,480 cd/m^2^, respectively.^9^ In addition to device efficiencies, the molecular orientation of the naphthyridine-based emitters with various donor moieties and their effects have been reported scarcely. The molecular orientations in the emitting layer are well-known to determine the outcoupling efficiency of OLEDs.^20,21^ The design of emitters taking into account the molecular orientation may allow to better predict device performance, which is important for the advancement of OLED technologies.

Inspired by the results of the recent investigations of naphthyridine derivatives, with the aim of investigation of the effect of the chemical modification on the properties, we designed four new naphthyridine-based electroactive organic compounds with donor and acceptor fragments linked via a phenyl bridge. The selection of an electron-accepting moiety was based on the simplicity of the synthesis and chemical modification possibility of the naphthyridine moiety. The nature of donor moieties, such as dimethylacridan, carbazole, phenothiazine, and phenoxazine in D–A molecular structures, affects most of the properties of the materials such as the HOMO, optical bandgaps, photoluminescence spectra, etc. In particular, a phenothiazine-based derivative was found to be the favorable TADF emitter among the synthesized four derivatives due to its highest photoluminescence quantum yield (PLQY), efficient TADF property with a high reverse intersystem crossing (rISC) rate constant of ca. 1.46 × 10^5^ s^–1^, and favorable horizontal molecular orientation. By incorporation of a wide-bandgap host material, 9,9′-(2-(1-phenyl-1*H*-benzo[*d*]imidazol-2-yl)-1,3-phenylene)bis(9*H*-carbazole) (*o*-DibzBz), a high-performance green TADF-based OLED with a maximum current efficiency (CE) of 58.6 cd/A, a maximum power efficiency (PE) of 57.1 lm/W, a maximum EQE of 16.4%, and Commission Internationale de L’éclairage (CIE) coordinates around (0.368, 0.569) was obtained. This PE value is clearly superior compared to those reported for other OLEDs based on naphthyridine derivatives.^10,13,15^ The nature of the electron-donating moiety was a crucial factor affecting the molecular orientation in the neat films, which was investigated by angle-dependent photoluminescence (ADPL) measurements. The dimethylacridan derivative exhibited the lowest orientation order parameter (Θ_ADPL_) of 0.37, indicating that the emissive molecules remained perpendicular with respect to the substrate. Meanwhile, the phenothiazine derivative exhibited the highest Θ_ADPL_ of 0.74, indicating that the emissive molecules aligned horizontally along the substrate. The molecular orientation was further supported by investigation on the molecular crystallization measuring grazing-incidence small-angle X-ray scattering (GIWAXS).^19^ Among the four studied compounds, the phenothiazine derivative exhibited a favorable scattering signal in the out-of-plane direction and minor crystalline issues, which benefit the carrier transport and device performance.

## 2 Experimental Section

### 2.1 Instrumentation

#### 2.1.1 Nuclear Magnetic Resonance (NMR) Spectroscopy

^1^H and ^13^C spectra were recorded by a Bruker Avance III apparatus (400 and 101 MHz). The samples were prepared by dissolving ca. 20 mg of a compound in 1 mL of deuterated chloroform (CDCl_3_) or dimethyl sulfoxide (DMSO-*d*_6_). Hydrogen nuclei ^1^H were excited using the frequency of 400 MHz. The data are presented as chemical shifts (δ) in ppm (in parentheses: multiplicity, integration, coupling constant). For attenuated total reflectance infrared spectroscopy (ATR-IR), IR spectra were recorded by using a Vertex 70 Bruker spectrometer equipped with an ATR attachment with a diamond crystal over frequencies of 600–3500 cm^–1^ with a resolution of 5 cm^–1^ over 32 scans. IR spectra are presented as a function of transparency (*T*) expressed in percent (%) against the wavenumber (*v*) expressed in cm^–1^. For mass spectrometry, mass spectra were obtained on a Waters ZQ 2000 mass spectrometer. Elemental analysis was performed with an Exeter analytical CE-440 elemental analyzer. For UV–vis absorption spectroscopy, absorption spectra of the dilute solutions (10^–4^–10^–5^ moL/L) and thin films of the compounds were recorded under ambient conditions with a PerkinElmer Lambda 25 spectrophotometer. For photoluminescence (PL) spectroscopy, fluorescence spectra of thin films and dilute solutions (10^–4^–10^–5^ moL/L) of the compounds were recorded at room temperature with a luminescence spectrometer Edinburgh Instruments FLS980. PL quantum yields of the solutions and thin films were measured using an integrating sphere. Phosphorescence spectra were recorded at 77 K. Differential scanning calorimetry (DSC) measurements were carried out using a TA Instruments Q2000 thermosystem. The samples were examined at a heating/cooling rate of 10 °C/min under a nitrogen atmosphere. Thermogravimetric analysis (TGA) was performed under a nitrogen atmosphere on a TA Instruments Q50 analyzer. The heating rate was 20 °C/min. Cyclic voltammetry measurements were performed by using a glassy carbon working electrode (a disk with a diameter of 2 mm) in a three-electrode cell of an Autolab-type potentiostat–galvanostat. The measurements were carried out for the solutions in dry dichloromethane containing 0.1 M tetrabutylammonium hexafluorophosphate at 25 °C; the scan rate was 50 mV/s, while the sample concentration was 10^–3^ M. The potentials were measured against silver as a quasi-reference electrode. A platinum wire was used as a counter electrode. The potentials were calibrated with the standard ferrocene/ferrocenium (Fc/Fc^+^) redox system.^20^ Ionization energy (IE) was calculated by employing the following formula 1:^21,22^
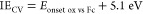
1

#### 2.1.2 Device Fabrication

OLEDs were fabricated by depositing the organic and metal layer in a thermal evaporator under 1 × 10^–6^ Torr onto patterned ITO glass substrates, which were pretreated by O_2_ plasma cleaning. Afterward, the OLEDs were transferred into a glovebox with a pure N_2_ environment, encapsulated by coverglass with UV-epoxy, and cured under UV radiation. Device performances of OLEDs, including current density–luminance–voltage characteristics *J*–*L*–*V*, EQE, PE and CE, EL spectra, and CIE 1931 coordinates, were measured by a spectrometer (Minolta CS-1000) under various electrical driving by a source meter (Keithley 2400). The setup of PLQY measurement consisted of a xenon lamp and a monochromator (Horiba, iHR320), an integrating sphere (Quanta-φ manual Rev C F-3029), a monochromator (Horiba, iHR320), a photomultiplier tube (Hamamatsu, PMT), and software (FluorEssence). The setup of TrPL measurement consisted of a 355 nm Nd-YAG picosecond pulse laser (PicoQuant VisUV), a monochromator (Horiba, iHR320), and a photomultiplier tube (Hamamatsu, PMT). The setup of TrEL measurement consisted of a function generator (Agilent 33500B), a source meter (Keithley 2400), a photomultiplier (Hamamatsu H6780-20), and an oscilloscope (Tektronix TDS2004C).

#### 2.1.3 Computational Methods

The ground-state geometries were optimized by using the B3LYP (Becke three-parameters hybrid functional with Lee–Yang–Perdew correlation)^23^ functional at the 6-31G(d,p) level in vacuum with the Gaussian^25^ program. First, equilibrium conformer search at the ground state was performed by using the MMFF (molecular mechanics force fields) method, and then, this geometry was used for further optimization. The vertical singlet and triplet energy values were calculated by using the energy values at the corresponding excited-state geometry. The time-dependent DFT (TD-DFT) calculations were carried out with the Gaussian 16 software package. Molecular orbitals were visualized by using GaussView.

### 2.2 Materials

2-Amino-3-formylpyridine, 4-bromoacetophenone, palladium(0) tetrakis(triphenylphosphine), phenothiazine, phenoxazine, sodium *tert*-butoxide, *tert*-butylchloride, zinc chloride (purchased from Aldrich), 3,7-di-*tert*-butyldimethylacridane (purchased from FMTC), sodium sulfate, and sodium hydroxide (purchased from Euro Chemicals) were used as received. Thin-layer chromatography was performed by using TLC plates covered with a silica gel matrix on aluminum backing (purchased from Aldrich). 3,6-Di-*tert*-butyl-carbazole was synthesized according to the reported procedure.^25^

#### 2.2.1 2-(4-Bromophenyl)-1,8-naphthyridine (*p*-NPBr)

2-Amino-3-formylpyridine (4 g, 0.0328 mol), 7.181 g (0.03608 mol) of 4-bromacetophenone, and 2 g of NaOH were dissolved in isopropanol (75 mL). The reaction was carried out at 83 °C for 24 h in an inert atmosphere. After the completion of the reaction, the mixture was cooled down and poured into distilled water. The yellow precipitate was filtered and washed additionally with distilled water before drying. The yield of yellow powder was 7.857 g (83%). MM 285.14 g/mol. ^1^H NMR (400 MHz, DMSO): δ 9.12 (d, *J* = 3.7 Hz, 1H), 8.59 (d, *J* = 8.5 Hz, 1H), 8.50 (d, *J* = 8.0 Hz, 1H), 8.30 (d, *J* = 8.0 Hz, 3H), 7.80 (d, *J* = 7.8 Hz, 2H), 7.65 (dd, *J* = 8.0, 4.2 Hz, 1H).

The following general procedure was used for the synthesis of target naphthyridine-based compounds. *p*-NPBr (7 mmol), the donor fragment (7 mmol), and Na *t*-BuO (35 mmol) were placed into a Schlenk flask and purged with nitrogen/evacuated in 3 cycles before adding toluene (30 mL) and palladium(0) tetrakis(triphenylphosphine) (0.3 mmol). The reaction mixture was refluxed overnight. After cooling to ambient temperature, it was poured into icy water. The aqueous phase was extracted with DCM (3 × 50 mL), the combined organic phases were dried over sodium sulfate and filtered, and then, the solvent was removed. The residue was purified by column chromatography on silica using an ethylacetate:*n*-hexane mixture (1:5) as an eluent.

#### 2.2.2 10-(4-(1,8-Naphthyridin-2-yl)phenyl)-3,6-di-*tert*-butyl-9*H*-carbazole (**EV1**)

The yield of yellow crystals was 1.428 g (42%.) MM = 483.65 g/mol. M.p. 270–272 °C. ^1^H NMR (400 MHz, DMSO): δ 9.15 (d, *J* = 2.8 Hz, 1H), 8.63 (t, *J* = 7.7 Hz. 3H), 8.54 (d, *J* = 7.8 Hz, 1H), 8.42 (d, *J* = 8.5 Hz, 1H), 8.33 (s, 2H), 7.86 (d, *J* = 8.4 Hz, 2H), 7.67 (dd, *J* = 8.0, 4.3 Hz, 1H), 7.51 (dd, *J* = 22.4; 8.7 Hz, 4H), 1.44 (s, 18H). ^13^C NMR (101 MHz, DMSO): δ 154.62, 144.01, 142.28, 133.25, 130.23, 128.95, 127.67, 126.47, 123.91, 120.37, 120.14, 109.82, 35.01, 34.81, 32.34. MS (ES^+^): *m*/*z* 484 [(M + H)]^+^. ATR-IR (solid state on ATR, cm^–1^): 3054 (C–H, ar.), 2959 (C–H, aliph.), 1597, 1471 (C=C, ar.). 1422 (C–H, aliph.), 1359, 1082, 1037 (C–N), 881, 840 (C–H, ar.). Elemental analysis for C_34_H_33_N_3_, % calc.: C, 84.43; H, 6.88; N, 8.69. % Found: C, 83.39; H, 6.86; N, 8.71.

#### 2.2.3 10-(4-(1,8-Naphthyridin-2-yl)phenyl)-3,7-di-*tert*-butyl-9,9-dimethyl-9,10-dihydroacridine (**EV2**)

The yield of yellowish crystals was 0.528 g (29%). MM 525.73 g/mol. M.p. 330–332 °C. ^1^H NMR (400 MHz, CDCl_3_): δ 9.12–9.08 (m, 1H), 8.48 (d, *J* = 8.3 Hz, 2H), 8.25 (d, *J* = 8.5 Hz, 1H), 8.17 (dd, *J* = 8.1, 1.6 Hz, 1H), 8.04 (d, *J* = 8.5 Hz, 1H), 7.43 (dt, *J* = 4.7, 3.2 Hz, 5H), 6.92 (dd, *J* = 8.6; 2.0 Hz, 2H), 6.22 (d, *J* = 8.6 Hz, 2H), 1.67 (s, 6H), 1.24 (s, 18H). ^13^C NMR (101 MHz, CDCl_3_): δ 159.67, 156.13, 154.04, 143.52, 142.96, 138.52, 138.08, 136.89, 131.72, 130.37, 129.58, 123.14, 122.27, 121.92, 119.78, 113.48, 36.47, 34.23, 31.62. MS (ES^+^): *m*/*z* 525 [M]^+^. ATR-IR (solid state on ATR, cm^–1^): 3048 (C–H, ar.), 2902, 2866 (C–H, aliph.), 1603, 1489 (C=C, ar.), 1411 (C–H, aliph.), 1362, 1018 (C–N), 891, 862 (C–H, ar.). Elemental analysis for C_37_H_39_N_3_, % calc.: C, 84.53; H, 7.48; N, 7.99. % Found: C, 83.49; H, 7.50; N, 7.97.

#### 2.2.4 10-(4-(1,8-Naphthyridin-2-yl)phenyl)-10*H*-phenothiazine (**EV3**)

The yield of yellow crystals was 1.645 g (58%). MM = 403.5 g/mol. M.p. 283–286 °C. ^1^H NMR (400 MHz, CDCl_3_): δ 9.09 (d, *J* = 2.5 Hz, 1H), 8.42 (d, *J* = 8.3 Hz, 2H), 8.23 (d, *J* = 8.5 Hz, 1H), 8.15 (d, *J* = 8.0 Hz, 1H), 7.99 (d, *J* = 8.5 Hz, 1H), 7.43 (dd, *J* = 8.1, 3.8 Hz, 3H), 7.03 (d, *J* = 7.4 Hz, 2H), 6.84 (dt, *J* = 23.6, 7.4 Hz, 4H), 6.42 (d, *J* = 8.0 Hz, 2H). ^13^C NMR (101 MHz, CDCl_3_): δ 159.45, 156.13, 154.04, 143.63, 138.02, 137.25, 136.86, 130.16, 128.82, 127.06, 123.18, 122.56, 121.88, 119.61, 117.88. MS (ES^+^): *m*/*z* 404 [M + H]^+^. ATR-IR (solid state on ATR, cm^–1^): 3058 (C–H, ar.), 1603, 1588, 1445 (C=C, ar.), 1308, 1016 (C–N), 865, 848 (C–H, ar.). Elemental analysis for C_26_H_17_N_3_S, % calc.: C, 77.39; H, 4.25; N, 10.41. % Found: C, 77.35; H, 4.23; N, 10.43.

#### 2.2.5 10-(4-(1,8-Naphthyridin-2-yl)phenyl)-10*H*-phenoxazine (**EV4**)

The yield of yellowish crystals was 1.48 g (54%). MM = 387.43 g/mol. M.p. 220–222 °C. ^1^H NMR (400 MHz, CDCl_3_): δ 9.06 (s, 1H), 8.43 (d, *J* = 8.3 Hz, 2H), 8.18 (t, *J* = 13.2 Hz, 1H), 8.12 (d, *J* = 7.8 Hz, 1H), 7.96 (d, *J* = 8.5 Hz, 1H), 7.40 (d, *J* = 8.2 Hz, 3H), 6.55 (ddd, *J* = 21.3, 15.9, 7.2 Hz, 6H), 5.93 (d, *J* = 7.7 Hz, 2H). ^13^C NMR (101 MHz, CDCl_3_): δ 158.16, 155.01, 153.05, 142.88, 139.68, 137.61, 137.13, 135.84, 133.04, 130.17, 129.56, 122.27, 121.05, 120.83, 120.45, 118.59, 114.47, 112.31. MS (ES^+^): *m*/*z* 388 [(M + H)]^+^. ATR-IR (solid state on ATR, cm^–1^): 3060, 3032 (C–H, ar.), 1597, 1484 (C=C, ar.), 1333 (C–N), 1265 (C–O), 1207, 1033, 1017 (C–N), 855, 739 (C–H, ar.). Elemental analysis for C_26_H_17_N_3_O, % calc.: C, 80.60; H, 4.42; N, 10.85. % Found: C, 80.55; H, 4.44; N, 10.83.

## 3 Results and Discussion

### 3.1 Synthesis and Thermal Properties

**Scheme 1:**
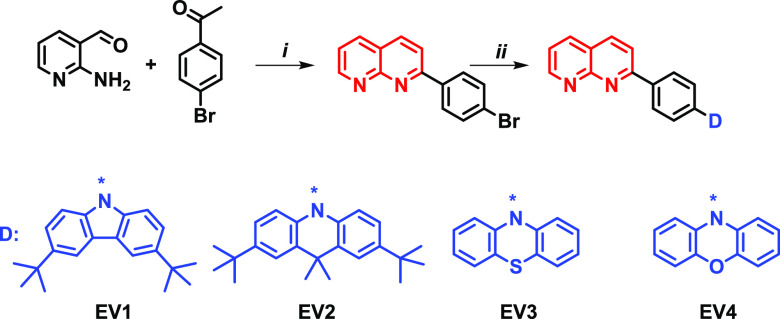
Synthesis Route to the Target Naphthyridine Derivatives (i) NaOH, isopropanol, 83 °C, 24 h; (ii) respective donor fragment, Na *t*-BuO, palladium(0) tetrakis(triphenylphosphine), toluene, reflux, 24 h.

The synthesis route to the target naphthyridine derivatives **EV1**–**4** is presented in Scheme 1. The naphthyridine moiety was formed by an environmentally friendly Friedländher cyclization reaction that allows to reach yields of 83%.^11^ The various donor moieties were attached by Pd-catalyzed Buchwald–Hartwig cross-coupling reaction.^26^ Naphthyridine-based compounds **EV1**–**4** were conveniently synthesized in moderate yields of 29–54%. The chemical structures of the target compounds were confirmed by ^1^H NMR (Figure S1), attenuated total reflectance Fourier transform infrared spectroscopy (ATR-FTIR), mass spectrometry, and elemental analysis.

The thermal properties of the naphthyridine derivatives **EV1**–**4** were investigated by thermogravimetric analysis (TGA) and differential scanning calorimetry (DSC) (Figures S2 and S3) The resulting data are presented in Table 1. Since the target compounds were isolated as crystalline compounds, the melting transitions of the samples **EV1**–**4** were detected during the first heating scans of the DSC measurements. Glass transitions were detected during the 2nd heating scans in the range of 80 to 137 °C for all naphthyridine derivatives, except **EV2**. In addition to high glass transition temperatures, high 5% weight loss temperatures of compounds **EV1**–**4** exceeding 360 °C were detected (Table S1).

**Table 1:** Photophysical Characteristics of Compounds **EV1**–**4**^a^

	toluene solution/thin film					
compound	λ_Abs_, nm	λ_FL_, nm	*S*_1_, eV	*T*_1_, eV	Δ*E*_ST_, eV	PLQY, %
**EV1**	-/295, 373	440/486	3.06	2.71	0.35	66/24
**EV2**	-/282, 322, 405	528/545	2.83	2.65	0.18	15/5
**EV3**	-/274, 326, 385	438, 576/531	2.88	2.60	0.28	7/1
**EV4**	-/274, 326, 412	529/561	2.81	2.68	0.13	15/6

a λ_Abs_ are wavelengths of absorption maxima; λ_FL_ are wavelengths of emission maxima; *T*_1_ is the triplet energy estimated as 1240/λ_PH_; Δ*E*_ST_ = *S*_1_ – *T*_1_.

### 3.2 Theoretical Calculations and Electrochemical Properties

The geometric structures and electronic transition characteristics of compounds **EV1**–**4** were investigated using density functional theory (DFT). The ground-state (S_0_) geometry was initially optimized at the B3LYP/6-31G(d,p) level in the gas phase, and then, the natural transition orbitals in the excited state S_1_ were generated using time-dependent DFT (TD-DFT) at the same level. The optimized geometry, transition energies, and natural transition orbitals of singlet excited states are shown in Figure S4.

**Figure 1:**
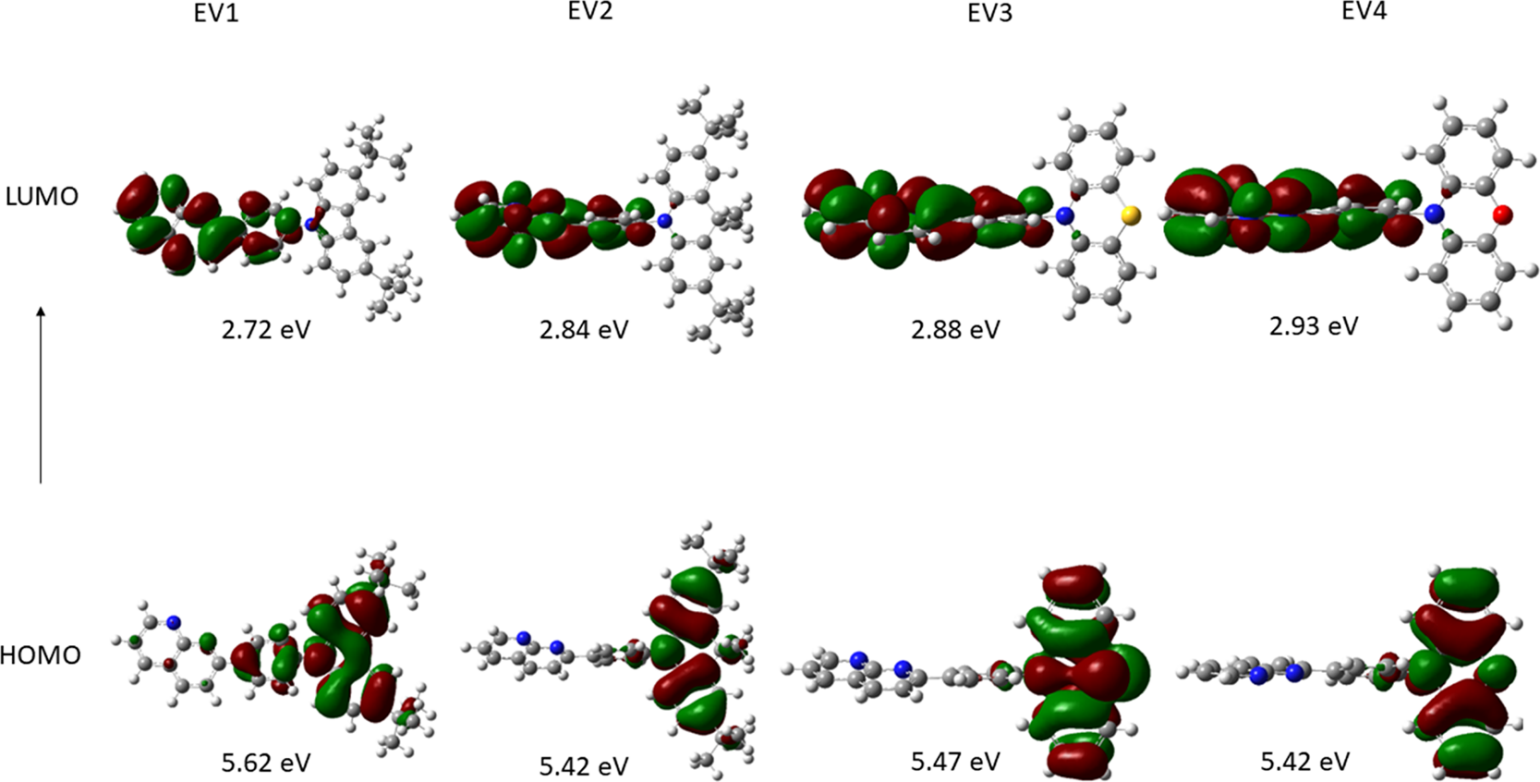
HOMO and LUMO visualizations of naphthyridine derivatives.

The lowest unoccupied molecular orbitals (LUMO) are located on the electron-accepting naphthyridine moiety, whereas the highest occupied molecular orbitals (HOMO) are mainly localized on the electron-donating 3,6-di-*tert-*butyl-carbazole (**EV1**), dimethylacridan (**EV2**), phenothiazine (**EV3**), and phenoxazine (**EV4**) moieties (Figure 1).

The naphthyridine moiety and donor substituents of compounds **EV1**–**4** are not planarized but have different dihedral angles between the fragments. The benzene ring as a π-bridge is planar with the naphthyridine moiety and is positioned with dihedral angles of 15–17°. The electron-donating 3,6-di-*tert*-butyl-carbazole fragment of **EV1** is positioned with a dihedral angle of 50° resulting in a more planar geometry at the ground state. Meanwhile, dimethylacridan (**EV2**), phenothiazine (**EV3**), and phenoxazine (**EV4**) moieties are positioned with dihedral angles of 89.7, 82, and 85.7°, respectively. Nevertheless, the dihedral angles between the D and A moieties at the S_1_ state were found to be close to 90° for **EV1**–**4** (Figure S4). The differences in dihedral angles of **EV1** at ground and excited states may result in larger conformational changes with a higher relaxation energy.

As the lowest excited S_1_ states are dominated by the transitions HONTO→LUNTO ICT, mainly due to perpendicular geometries (Figure S4), this clear separation of the frontier molecular orbitals of compounds **EV2**–**4** leads to very small oscillator strength values of ca. 0.0, enabling efficient rISC that is a prerequisite for efficient TADF.

Cyclic voltammetry (CV) was used to investigate the electrochemical properties of the naphthyridine derivatives (**EV1**–**4**). The oxidation of the compounds was found to be reversible up to 1.25 V (Figure S5). Ionization energy values (IE_CV_) of the compounds were calculated on the basis of the onset voltages of the oxidation. The IE_CV_ values were found to depend on donor moieties of the D–A compounds. They were found to be of 5.71 eV for **EV1**, 5.56 eV for **EV2**, 5.51 eV for **EV3**, and 5.46 eV for **EV4**.

### 3.3 Photophysical Properties

**Figure 2:**
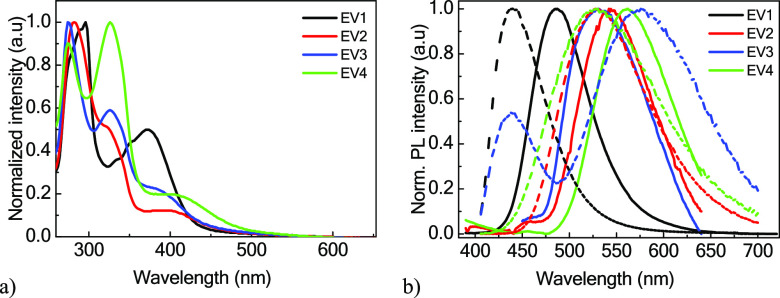
(a) UV/vis absorption spectra of the solid-state samples and (b) and emission spectra of the solid samples (solid) and dilute toluene solutions (dashed) of naphthyridine derivatives **EV1**–**4**. λ_ex_ = 330 nm.

UV/vis and photoluminescence (PL) spectra of the solid samples of the naphthyridine derivatives (**EV1**–**4**) are shown in Figure 2a,b. The higher-energy absorption bands of compounds **EV1**–**4**, which range up to 370 nm, can be assigned to the locally excited π→π* transition of donor moieties (carbazole, dimethylacridan, phenothiazine, and phenoxazine).^19,27−29^ The difference of the positions of the band peaks is apparently due to the different electron-donating abilities of the donor moieties. The lower-energy absorption bands above 370 nm correspond to the intermolecular charge transfer (ICT) from the donor moieties to the naphthyridine acceptor moiety.

PL intensity peaks (Table 1) were redshifted upon an increase in the electron-donating abilities of the donor moieties. The emission intensity peaks of the toluene solutions of **EV1**–**4** were observed at 440, 528, 576, and 529 nm. The peak emission intensity of **EV3** observed at 440 nm might be attributed to the emission from the phenothiazine moiety (Figure S6). The PL spectra of the toluene solutions of **EV1**, **EV2**, and **EV4** were blueshifted compared to those of the solid samples, with the highest PLQY values reaching 66% (**EV1**). The redshift of the PL spectrum of toluene solution of **EV3** compared to that of the solid sample can be attributed to the aggregation-induced delayed fluorescence in the solid state (Figures S6 and S7). Aggregation-induced emission enhancement was tested for the dispersions of **EV3** in THF/water mixtures with different water contents (Figure S7). As the concentration of water increased up to 60%, the emission profile with emission peaks around 540–550 nm became similar to that of the solid-state sample. The PL bands of the solid samples of compounds **EV1**–**4** were broad and structure-less, as shown in Figure 2b. The emission efficiencies of the neat films were found to be not very high. The highest PLQY value of 24% was observed for **EV1**.

**Figure 3:**
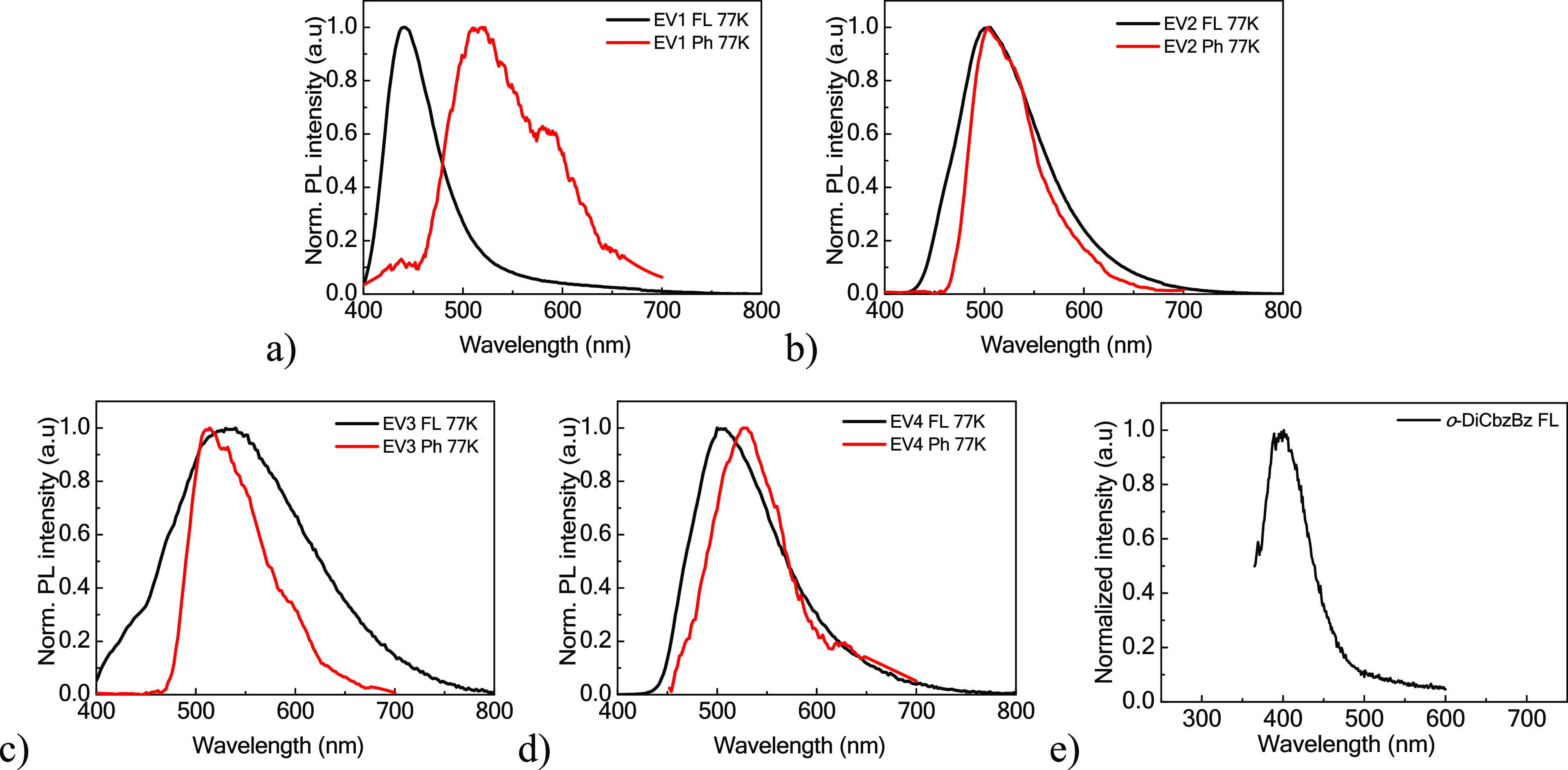
(a–d) Photoluminescence and phosphorescence (Ph) spectra of compounds of the molecular dispersions of **EV1**–**4** in the polymeric Zeonex480 matrix recorded at 77 K. Ph spectra were recorded with a microsecond lamp with a delay of 1 μs. (e) PL spectrum of a neat host *o*-DiCbzBz film.

To determine the triplet energies and singlet–triplet splittings (Δ*E*_ST_) of compounds **EV1**–**4**, 10% solid solutions of emitters in the Zeonex480 polymeric matrix were prepared, and PL measurements were performed. The PL and phosphorescence (Ph) spectra of the compounds (**EV1**–**4**) were recorded at 77 K (Figure 3a–d). The established singlet and triplet energy levels (Table 1) were found to be close for **EV2**–**4** (Δ*E*_ST_ = 0.13–0.28 eV), confirming the possibility of efficient direct and reverse intersystem crossing from T_1_ to S_1_ and demonstrating the potential characteristics of TADF. The larger Δ*E*_ST_ of **EV1** might result from a flatter conformation of the molecules dispersed in the Zeonex matrix.

**Figure 4:**
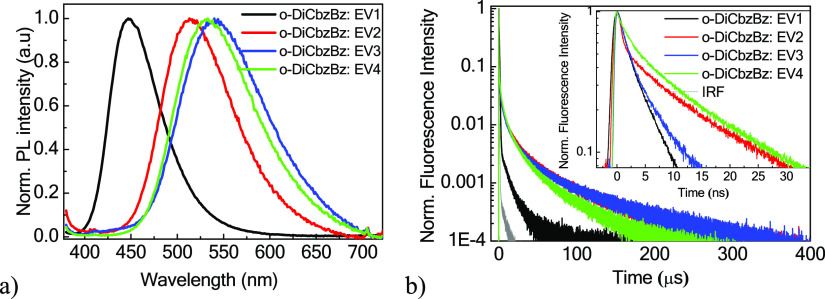
(a) Normalized PL spectra and (b) TrPL signals of the films of **EV1**–**4**-doped *o*-DiCbzBz in a microsecond window. The inset of (b) shows the TrPL signal in a nanosecond window.

To examine the TADF characteristics of naphthyridine derivatives, 10 vol % molecular dispersions of emitters (**EV1**–**4**) in the host *o*-DiCbzBz^30^ were prepared, and PL measurements were performed including time-resolved photoluminescence measurements (Figure 4). The emission bands of the films of the samples of doped **EV1**, **EV2**, and **EV4** were blueshifted compared to their neat film PL bands. This observation can be attributed to weaker intermolecular interactions in the Zeonex matrix. The PL decay curves of the films of the host–guest systems exhibited multiexponential behaviors. Their characteristics are summarized in Table 2. The fast component in the nanosecond window can be attributed to the prompt emission from the singlet state, while the delayed components in the μs window originated from the transition from T_1_ to S1 via rISC.^31^ The PLQY (Φ) values of the films of the host–guest systems were also measured. They are summarized in Table 2. The PLQY values are considerably higher compared to those of the solutions and neat films except **EV1**. The singlet radiative (*k*_r_), ISC (*k*_ISC_), and rISC (*k*_rISC_) were obtained by calculations from the PLQY values (Figure S8) and emission lifetimes of prompt (τ_p_) and delayed components (τ_d_) of the films of the host–guest systems (Figure 4 and Table 2).^32^ The prompt (Φ_F_) and delayed (Φ_TADF_) components of PLQY were further separated and obtained by integrating the intensity in the ranges of 0–1 and 1–400 μs of TrPL signals, respectively. The detailed calculations are presented in the Supporting Information (Figure S9). The rISC rate of the carbazole-containing naphthyridine derivative **EV1** was found to be the smallest one of 1.33 × 10^4^ (s^–1^). This observation can be attributed to the largest Δ*E*_ST_ of 0.35 eV among the four derivatives, which implies the lowest TADF efficiency of **EV1**. The films of the host–guest systems containing **EV2**, **EV3,** and **EV4** exhibited *k*_rISC_ in the range of 4.2 × 10^4^–1.4 × 10^5^ (s^–1^) with delayed emission lifetimes τ_d_ of 17–22 μs, which are similar to those of the reported naphthyridine-based emitters.^2,15^ The favorable conformations in the solid samples of host–guest systems apparently resulted in stronger intramolecular charge transfer with smaller Δ*E*_ST_. Hence, **EV2**, **EV3**, and **EV4** have potential for the application in TADF-based OLEDs owing to their comprehensively high PLQY values and high rISC rates.

**Table 2:** Fitting Results^a^ of PL Decay Curves of the Films of **EV1**–**4** (10%) Doped in *o*-DiCbzBz

	λ_PL_, nm	Φ, %	Φ_F_, %	Φ_TADF_, %	τ_p_, ns	*k*_r_, s^–1^	*k*_ISC_, s^–1^	τ_d_, μs	*k*_rISC_, s^–1^	*k*_nt_^T^, s^–1^
**EV1**	449	38	35	3	4.4	7.98 × 10^7^	1.47 × 10^8^	10.5	1.33 × 10^4^	9.06 × 10^4^
**EV2**	513	29	14	15	11.2	1.26 × 10^7^	7.67 × 10^7^	22.2	5.66 × 10^4^	3.71 × 10^4^
**EV3**	539	61	29	32	5.6	5.23 × 10^7^	1.26 × 10^8^	21.1	1.46 × 10^5^	5.24 × 10^4^
**EV4**	531	58	36	22	10.7	3.40 × 10^7^	5.94 × 10^7^	17.8	4.26 × 10^4^	2.95 × 10^4^

a The calculations are presented in the SI.

### 3.4 Electroluminescence

**Figure 5:**
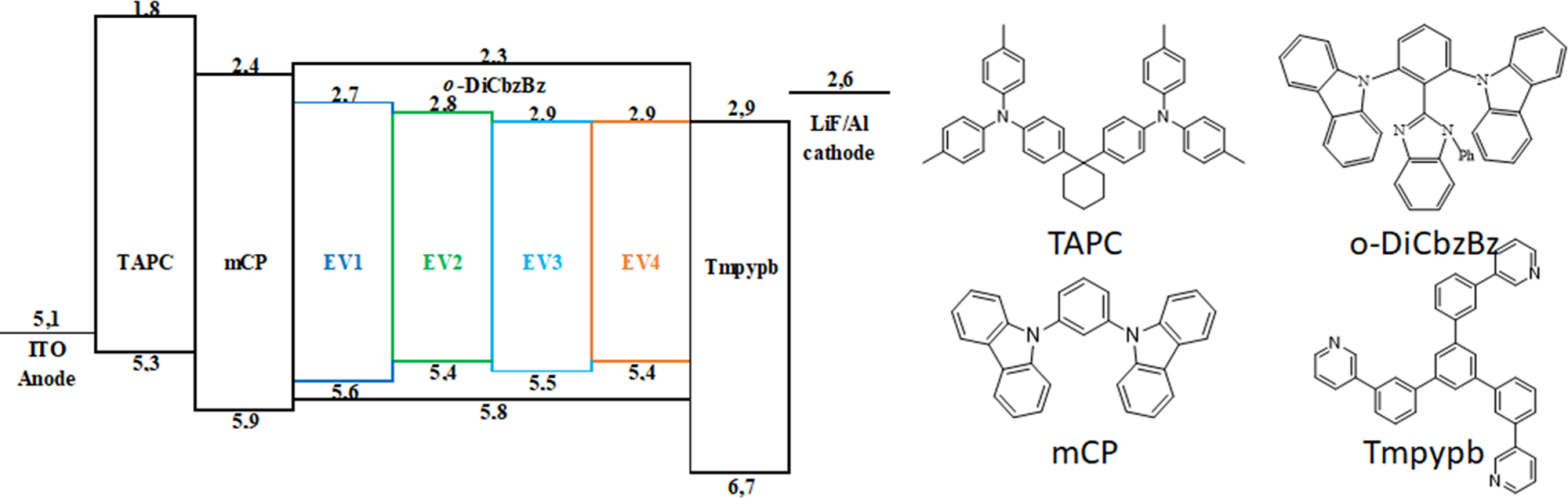
Device structure with the energy diagram and molecular structures of each layer.

To study the performance of naphthyridine derivatives in TADF-based OLEDs, four emitters **EV1**–**EV4** were applied as dopant materials and *o*-DiCbzBz as the host material for the emitting layer (EML). The optimized device structure (Figure 5) was ITO/1,1-bis[(di-4-tolylamino)phenyl]cyclohexane (TAPC) (50 nm)/1,3-di(9*H*-carbazol-9-yl)benzene (mCP) (10 nm)/*o*-DiCbzBz:10% naphthyridine derivatives (30 nm)/1,3,5-tris(3-pyridyl-3-phenyl)benzene (TmpyPB) (45 nm)/LiF (0.8 nm)/Al (120 nm), where TAPC, mCP, and TmpyPB were the hole-transporting, hole-transporting, and electron-transporting materials, respectively. The dopant concentration of the EML was optimized in terms of EQE values as it is shown in Figure S10. The host material applied in this study (*o*-DiCbzBz) possesses the wide bandgap with a high triplet energy exceeding 3.0 eV, excellent horizontal molecular orientation, and bipolar charge-transporting properties, which make it applicable in OLEDs with emission from sky blue to orange.^19,24^

**Figure 6:**
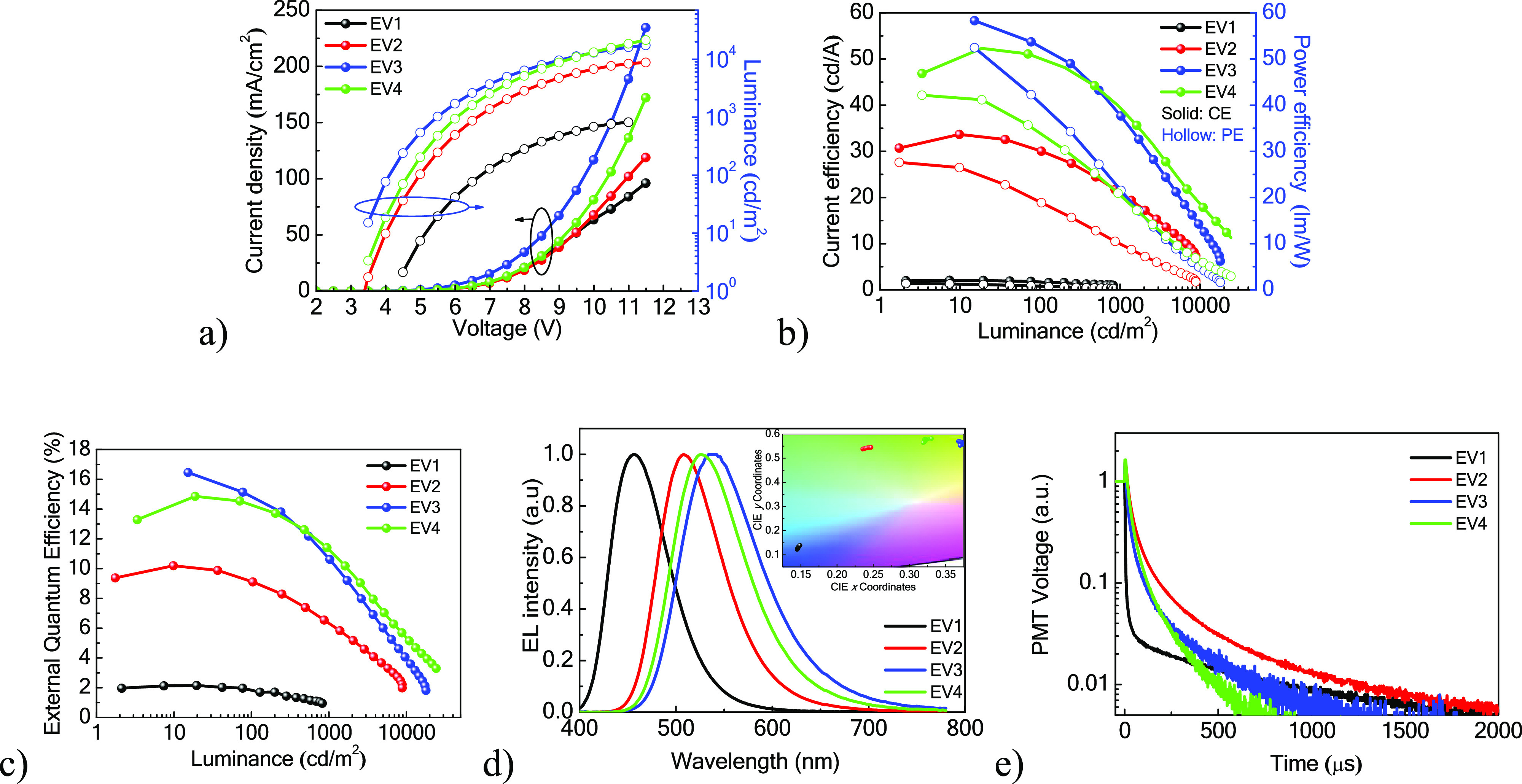
(a) *J*–*V*–*L*, (b) CE and PE–*L*, and (c) EQE–*L* plots; (d) EL spectra recorded at 5 V; (e) TrEL recorded at a constant current of 1 mA/cm^2^ followed by −9 V reversed bias at a 2000 μs window. The inset in (d) is CIE coordinates.

Current density–voltage–luminance (*J*–*V*–*L*), current efficiency (CE) and power efficiency (PE)–L, and EQE–L plots, electroluminescence (EL) spectra recorded at 5 V, and transient EL (TrEL) signals of TADF OLEDs are shown in Figure 6a–e. The characteristics of the devices are summarized in Table 3. The turn-on voltages at 1 cd/m^2^ of these four devices are directly related to their emitted photon energy, corresponding to their energy bandgap. A blue photon requires a high voltage to be excited. The driving voltage might be affected by several parameters such as the optical bandgap of the host and emitters, carrier trapping, carrier injection, carrier balance, etc.

**Table 3:** EL Characteristics of TADF OLEDs with the Device Structure ITO/TAPC (50 nm)/mCP (10 nm)/*o*-DiCbzBz:10% Emitter (30 nm)/TmpyPB (45 nm)/LiF (0.8 nm)/Al (120 nm)

emitter	driving voltage^a^ (V)	CIE^b^	EL peak^b^ (nm)	CE^c^(cd/A)	PE^c^(lm/W)	EQE^c^ (%)	Θ_ADPL_^d^	*S*_GIWAXS_^d^
**EV1**	4.2/4.4	(0.145, 0.127)	456	2.5/-	1.4/-	2.2/-	0.45	0.21
**EV2**	3.4/3.9	(0.236, 0.538)	508	33.7/20.1	27.6/9.8	10.1/6.3	0.37	0.16
**EV3**	2.9/3.8	(0.368, 0.569)	538	58.6/37.8	57.1/21.8	16.4/10.6	0.74	0.27
**EV4**	3.2/4.3	(0.321, 0.578)	526	52.3/39.4	42.1/20.4	14.8/11.2	0.62	0.20

a Recorded at 1 cd/m^2^ and 1 mA/cm^2^.

b Recorded at 5 V.

c Recorded at maximum and 1000 cd/m^2^.

d 10% solid solution of the emitter in *o*-DiCbzBz (30 nm).

As it is shown in Figure 6a, the **EV3**-based OLED had the lowest turn-on voltage of 1 cd/m^2^ and driving voltages at a *J* of 1 mA/cm^2^ among four devices. The **EV3**-based OLED exhibited the best characteristics among four devices: a maximum CE (CE_max_) value of 58.6 cd/A, a maximum PE (PE_max_) value of 57.1 lm/W, and an EQE_max_ value of 16.4%. These characteristics were observed due to the highest PLQY value (61.1%) and the efficient rISC of the film of the host–guest system (Table 3). The PE performance of the **EV3**-based TADF OLED was the best among the reported naphthyridine-based OLEDs.^10,13,15^ Taking the value of PLQY (Φ = 61.1%) and assuming that exciton recombination (*r*) and exciton utilization efficiency are 100%, the outcoupling efficiency of the TADF OLED was estimated to be 26.8%. This value is higher than the value based on the conventional assumption that the outcoupling efficiency is 20%. The results of ADPL and GIWAXS measurements discussed later support this value. Although the **EV4**-based OLED had a slightly lower EQE_max_ compared to the **EV3**-based one, the better efficiency roll-off resulted in a higher EQE observed at a higher luminance. The inferior device performance of the **EV2**-based OLED can be ascribed to a lower PLQY with reasonable rISC rates. The **EV1**-based OLED showed a low device efficiency due to the lowest PLQY value and the insufficient rISC that resulted in the lowest triplet contribution (Table 2).

EL spectra of the devices are strongly related to the nature of electron-donating groups of the derivatives of naphthyridine (Figure 6d). The **EV1**-based device exhibited a blue emission with the EL intensity peak at 456 nm recorded at 5 V. This observation can apparently be attributed to the conformational disorder and strong ICT restrictions.^33^ Multicolor EL of TADF OLEDs based on **EV2**, **EV3**, and **EV4** from green to yellowish was observed with peaks at 508, 538, and 526 nm, respectively. The EL spectrum of the **EV3**-based TADF OLED corresponds to the aggregation-induced delayed fluorescence emission spectra of the dispersions in THF/water mixtures (Figure S7) observed at 530–546 nm. Transient electroluminescence (TrEL) analysis (Figure 6e) showed clear delayed fluorescence signals in the 2000 μs window, attributed to TADF for OLEDs based on compounds **EV1**–**4**. The device based on **EV1** had the lowest delayed emission ratio of less than 0.03, indicating low triplet exciton utilization. **EV2**–**4**-based devices showed much higher delayed emission contributions, indicating their efficient TADF properties.

**Figure 7:**
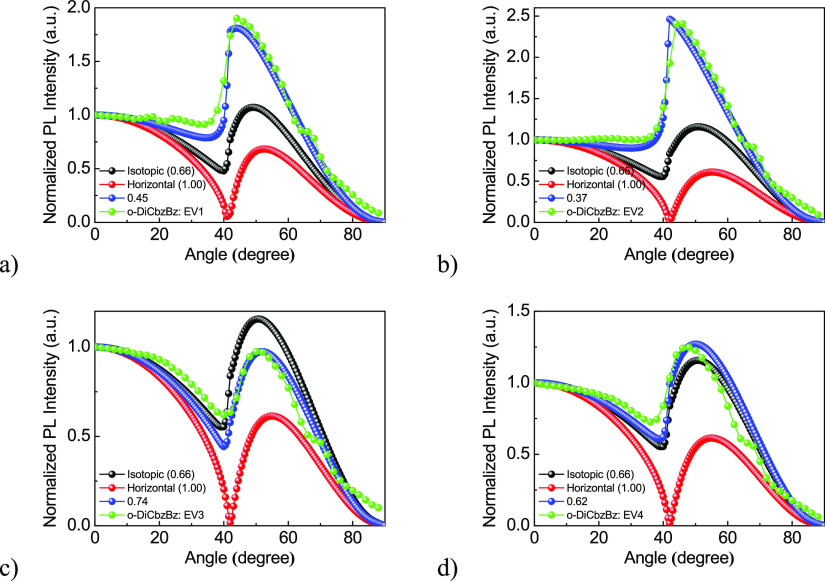
ADPL intensity and simulated orientation order parameter (Θ_ADPL_) of the molecular mixtures (a) *o*-DiCbzBz and **EV1**, (b) *o*-DiCbzBz and **EV2**, (c) *o*-DiCbzBz and **EV3**, and (d) *o*-DiCbzBz and **EV4**.

To investigate the optical transition dipole of emitters, ADPL measurements were performed for the aforementioned films (30 nm) of 10% solid solutions of **EV1**–**4** in the *o*-DiCbzBz host. A 325 nm laser was used to excite the mixtures. The ADPL monitored the emission intensity from emitters to realize the optical transition dipoles and molecular orientation of the emitters, as shown in Figure 7. Obviously, these four molecular mixtures exhibited different normalized ADPL intensity profiles, indicating that they had diverse molecular orientations resulting from the intrinsic emitter alignment and extrinsic interaction by the host arrangement.

By using a conventional wide-bandgap material, bis[2-(diphenylphosphino)phenyl]ether oxide (DPEPO), as the host matrix, the orientation factors were found to be smaller compared to those obtained with *o*-DiCbzBz (Figure S13). Hence, we used the host material *o*-DiCbzBz in this study. It was reported to have the function of promoting the horizontal molecular orientation of emitters.^34^ The horizontal Θ_ADPL_ of these mixtures and their simulation results with default Θ_ADPL_ values of 1.00 and 0.66 for the perfect horizontal and isotropic dipole orientation are also shown in Figure 7. The estimated horizontal Θ_ADPL_ values of the molecular mixtures of **EV1**–**4** and the host were found to be 0.37, 0.45, 0.74, and 0.62, respectively. Therefore, it is possible to conclude that most of the molecules of **EV1** and **EV2** sat on the substrate to form the vertical transition dipoles. Meanwhile, a great amount of the molecules of **EV3** reclined on the substrate to form the horizontal transition dipoles. The orientation of the molecules of **EV4** was found to be close to the isotropic one. The highest Θ_ADPL_ of *o*-DiCbzBz doped with **EV3** allows to predict the potential for high outcoupling efficiency, which is one of the reasons for the high efficiency of **EV3**-based OLEDs. The reported 1,5-naphthridine-based emitters^9,35^ of the EV series exhibited smaller Θ_ADPL_ values since their molecular structures are nonlinear and asymmetric, which leads to the higher freedom of molecular rotation during the film deposition.

**Figure 8:**
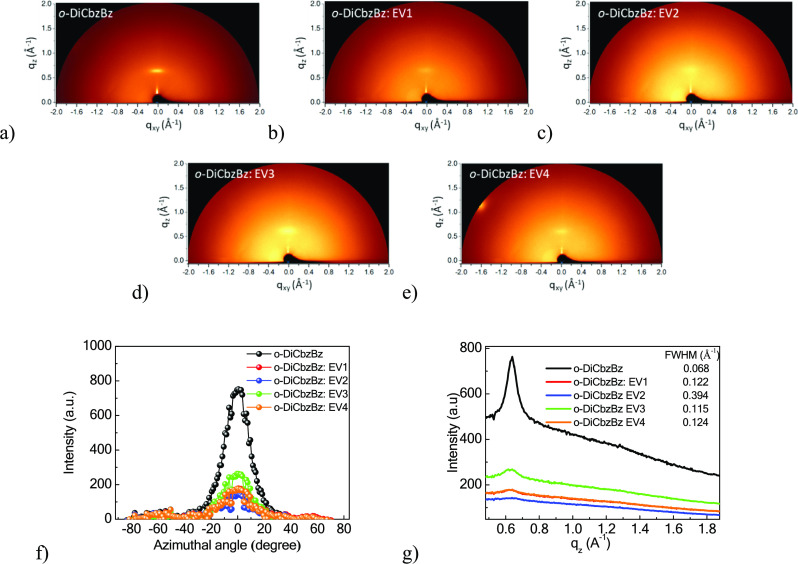
Two-dimensional GIWAXS patterns of (a) *o*-DiCbzBz, (b) *o*-DiCbzBz doped with **EV1**, (c) *o*-DiCbzBz doped with **EV2**, (d) *o*-DiCbzBz doped with **EV3**, and (e) *o*-DiCbzBz doped with **EV4**. Integral intensity of GIWAXS patterns of (f) different azimuthal angles at a *q_xy_* of 0.66 Å^–1^ and (g) different *q_z_* at an azimuthal angle of 0°.

In addition, a powerful GIWAXS measurement was used to further confirm the orientation of molecules in the molecular mixtures and also explore their crystalline properties.^36^ This analysis was used to evaluate the orientation of several organic molecules applied in OLEDs.^19^ The molecular mixtures and the pristine *o*-DiCbzBz host were deposited on silicon substrates for the GIWAXS measurement (Figure 8a–e). The neat film of *o*-DiCbzBz exhibited a striking π–π stacking-like signal (corresponding to a *d*-spacing of ∼9.5 Å) in the out-of-plane direction as shown in Figure 8a. A lamellar stacking scattering from lateral backbone-to-backbone separation was observed, corresponding to a *d*-spacing of ∼19 Å. Since the stronger π–π stacking-like and lamellar scattering signals were respectively displayed in the *q_z_* and *q_xy_* direction, the orientation of *o*-DiCbzBz could be claimed as a “face-on” one. When the naphthyridine-based luminophores (**EV1**–**4)** were doped into the *o*-DiCbzBz host matrix, the GIWAXS images exhibited some discrepancies as shown in Figure 8b–e. The π–π stacking-like and lamellar scattering signals became blurred and weak, indicating that dopants **EV1**–**4** disturb the host arrangement. In the case of the sample containing **EV3**, the relatively bright and clear scattering signals, similar to that of the *o*-DiCbzBz host, were observed. This observation shows that molecules of **EV3** are flexible to align with the host and show a favorable horizontal orientation.

To further quantify the 2D GIWAXS images, the intensities at various azimuthal angles (χ) of GIWAXS patterns were collected along a semicircular arc with *q_xy_* = 0.66 Å^–1^ radii (Figure 8f.) The orientation order parameters GIWAXS (*S*_GIWAXS_) of pristine *o*-DiCbzBz and of its molecular mixtures with **EV1**–**4** mixtures were calculated as 0.37, 0.21, 0.16, 0.27, and 0.20, respectively. The calculations were performed using eqs 1 and 2:
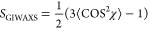
2
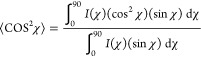
3

In general, the *S*_GIWAXS_ values range from −0.5 (for the perpendicular position with respect to the substrate) to 1 (for the molecules aligned horizontally along the substrate). For the totally random distribution, the *S*_GIWAXS_ value is 0. Here, the high *S*_GIWAXS_ of the pristine *o*-DiCbzBz thin film represents the horizontal molecular orientation, which is close to our previous results.^19^ The decreased *S*_GIWAXS_ value after introduction of **EV1**–**4** into the *o*-DiCbzBz host matrix expresses that the dopants **EV1**–**4** disturb the molecular packing of the host. Among the molecular mixtures, the system containing **EV3** exhibits the highest *S*_GIWAXS_ value of 0.27 approaching to 0.37 of the pristine thin film of *o*-DiCbzBz. This observation is explained by the dopant flexibility to align with the host orientation. The intensities of the profiles versus *q_z_* at *q_xy_* = 0 Å^–1^ (χ = 0°) are plotted in Figure 8g. They allow to identify the full width at half-maximum (FWHM) of the diffraction peaks and to evaluate the crystalline size using the Scherrer equation (eq 3):
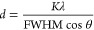
4where *d*, *K*, λ, and θ are the crystalline domain sizes, the dimensionless shape factor, the incident X-ray wavelength, and Bragg’s angle, respectively. The calculated FWHM values of the pristine *o*-DiCbzBz and its molecular mixtures with **EV1**–**4** are 0.068, 0.122, 0.394, 0.115, and 0.124 Å^–1^, respectively. The smaller FWHM value represents the larger crystalline domain size. Hence, it is evident that the host crystalline domain was reduced by introducing the dopants. Among the studied emitters, **EV3** shows the largest crystalline domain size. This observation confirms that the molecules of **EV3** are flexible to retain the largest host crystalline domain size. This may be beneficial to the charge carrier transport in **EV3**-based OLEDs, leading to the low driving power and high power efficiency.

## 4 Conclusions

Donor–acceptor-type derivatives of naphthyridine and dimethylacridan, carbazole, phenothiazine, or phenoxazine were obtained by two-step synthesis. The different strengths of electron-donating moieties influenced their HOMO values and optical bandgaps, resulting in different emissions from blue to green to yellowish green. The phenothiazine derivative was found to be the most efficient TADF emitter among the four derivatives because of its efficient reverse intersystem crossing, favorable photoluminescence quantum yield, and horizontal molecular orientation. The high-performance TADF OLED based on the derivative of naphthyridine and phenothiazine exhibited a maximum current efficiency of 58.6 cd/A, a record-high power efficiency of 57.1 lm/W, and an external quantum efficiency of 16.4% with color coordinates around (0.368, 0.569). The favorable horizontal orientation and crystalline domain size of the derivative of naphthyridine and phenothiazine in the emitting layer of OLEDs are supported by angle-dependent photoluminescence and grazing-incidence small-angle X-ray scattering studies.

## Supporting Information Available

^1^H NMR spectra of naphthyridine-based compounds (Figure S1); TGA and DSC thermograms of naphthyridine-based compounds (Figures S2 and S3); table of thermal characteristics (Table S1); figures of optimized geometries and natural transition orbitals of optimized structures (Figure S4); CV voltammograms (Figure S5); UV and PL spectra of **EV3** and phenothiazine toluene solutions (Figure S6); PL and TrEL spectra of **EV3** in THF/water mixtures with different water fractions (Figure S7); PLQY of thin films of **EV1**–**4** (10%) doped in *o*-DiCbzBz (Figure S8); optical diagram of **EV1**–**4** (10%) doped in *o*-DiCbzBz (Figure S9); figures of *J*–*V*–*L*, CE and PE–*L*, and EQE–*L* plots and normalized EL spectra recorded at 5 V (Figure S10); figure of TrPL signals of **EV1**–**4** in neat films in a 4 μs window (Figure S11); figures of normalized absorbance and normalized PL spectra of **EV1**–**EV4** dissolved in DCM, THF, and toluene (Figure S12); figures of the ADPL intensity and simulated orientation order parameter of **EV3** and **EV4** in a DPEPO matrix (Figure S13) (PDF)
