# Supplementary material for: Power Efficiency Enhancement of Organic Light-Emitting Diodes Due to the Favorable Horizontal Orientation of a Naphthyridine-Based Thermally Activated Delayed Fluorescence Luminophore
Source: ACS Appl Electron Mater. 2023 Jan 25;5(2):1013–23. doi: 10.1021/acsaelm.2c01529 (PMC9979784; doi:10.1021/acsaelm.2c01529)
Supplement: Supplementary file 1 — el2c01529_si_001.pdf [file el2c01529_si_001.pdf]

Supporting Information for

Power efficiency enhancement of OLEDs due to favourable horizontal orientation of naphthyridine-based TADF luminophore

Rasa Keruckiene[1], Eimantas Vijaikis[1], Chia-Hsun Chen[2], Bo-Yen Lin[3], Jing-Xiang Huang[2], Chun-Chieh Chu[2], Yi-Chung Dzeng[4], Chi Chen[4], Jiun-Haw Lee[2], Tien-Lung Chiu*[5], Simas Macionis[1], Jonas Keruckas[1], Rita Butkute[1], Juozas Vidas Grazulevicius[1]**

* Corresponding authors: Juozas Vidas Grazulevicius, juozas.grazulevicius@ktu.lt Department of Polymer Chemistry and Technology, Faculty of Chemical Technology, Kaunas University of Technology, K. Barsausko st. 59-450, Kaunas, Lithuania; Tien-Lung Chiu, tlchiu@saturn.yzu.edu.tw Department of Electrical Engineering, Yuan Ze University, Taiwan; Jiun-Haw Lee, jiunhawlee@ntu.edu.tw Graduate Institute of Photonics and Optoelectronics, National Taiwan University, Taipei 10617, Taiwan.

[1]Department of Polymer Chemistry and Technology, Kaunas University of Technology, K. Barsausko St. 59, LT-50254, Kaunas, Lithuania

[2]Graduate Institute of Photonics and Optoelectronics and Department of Electrical Engineering, Material Science and Engineering, and Physics, National Taiwan University, Taipei 10617, Taiwan

[3]Department of Opto-Electronic Engineering, National Dong Hwa University, Shoufeng, Hualien 974301, Taiwan.

[4]Research Center for Applied Sciences, Academia Sinica, Taipei 11529, Taiwan

[5]Department of Electrical Engineering, Yuan Ze University, Taiwan

Table of Contents

Figures and tables 2

References 13

Figures and tables

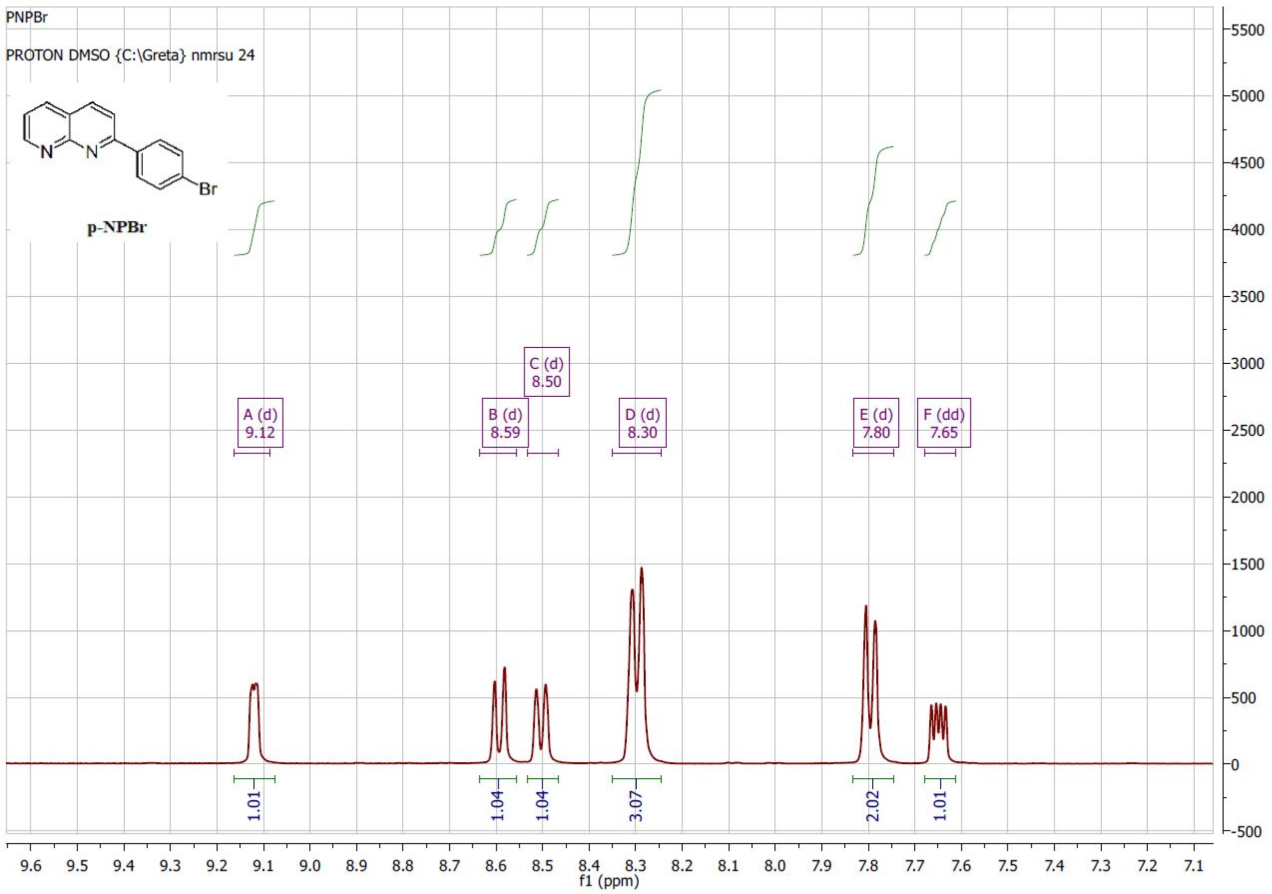

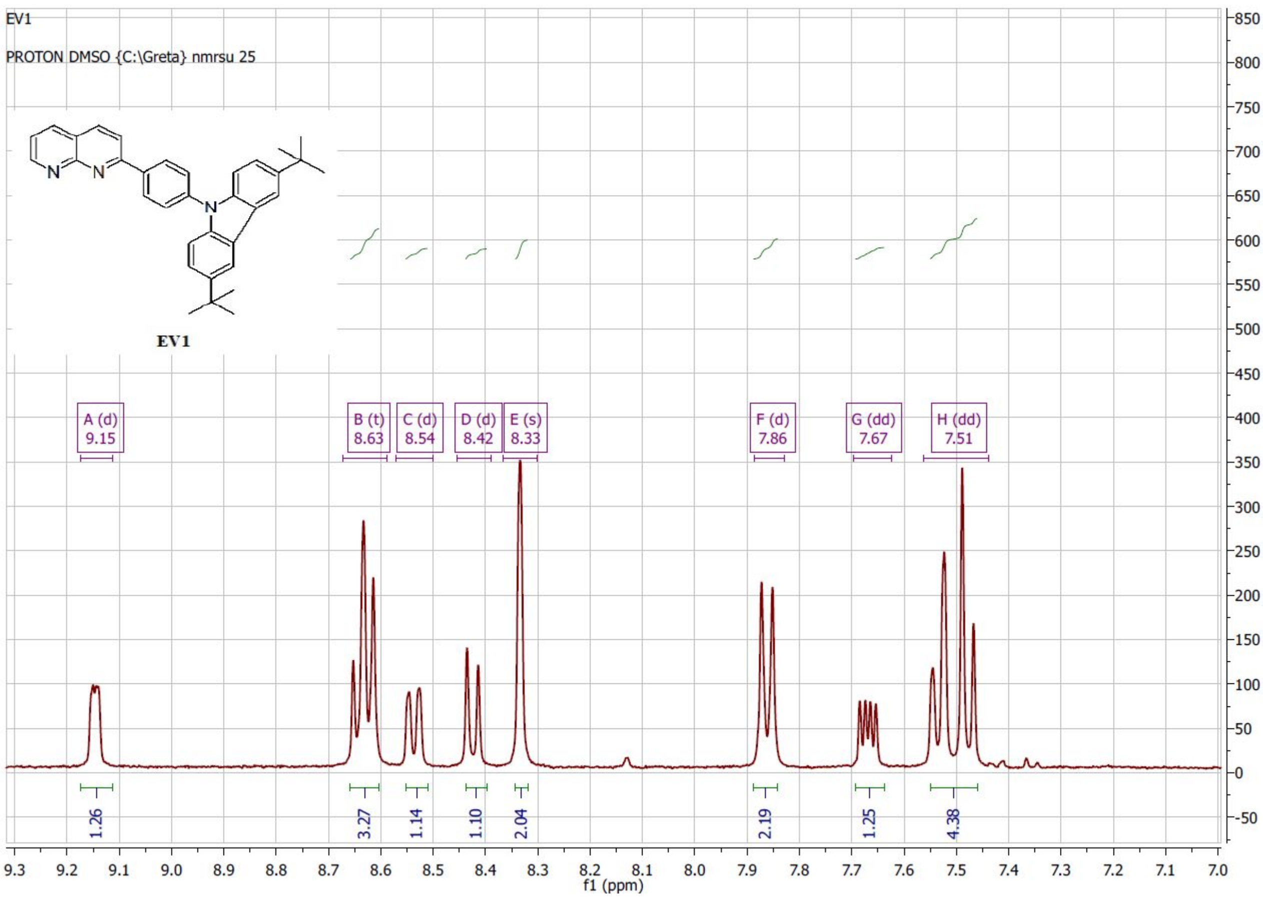
EV1
PROTON DMSO {C:\Greta} nmrsu 25
EV1
A (d) 9.15
B (t) 8.63
C (d) 8.54
D (d) 8.42
E (s) 8.33
F (d) 7.86
G (dd) 7.67
H (dd) 7.51
1.26
3.27
1.14
1.10
2.04
2.19
1.25
4.38
f1 (ppm)

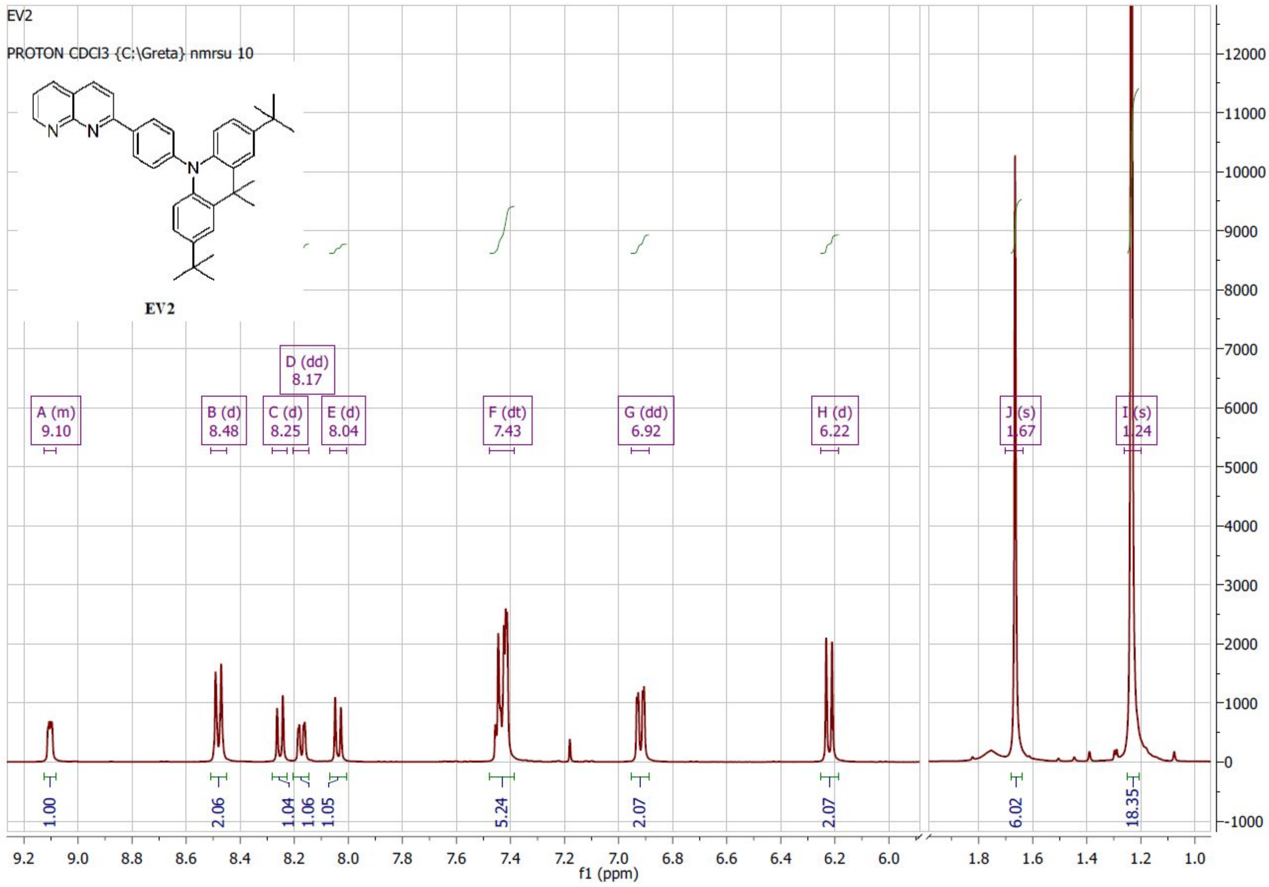
EV2
PROTON CDCl3 {C:\Greta} nmrsu 10
EV2
A (m) 9.10
B (d) 8.48
C (d) 8.25
D (dd) 8.17
E (d) 8.04
F (dt) 7.43
G (dd) 6.92
H (d) 6.22
J (s) 1.67
I (s) 1.24
1.00
2.06
1.04
1.06
1.05
5.24
2.07
2.07
6.02
18.35
f1 (ppm)

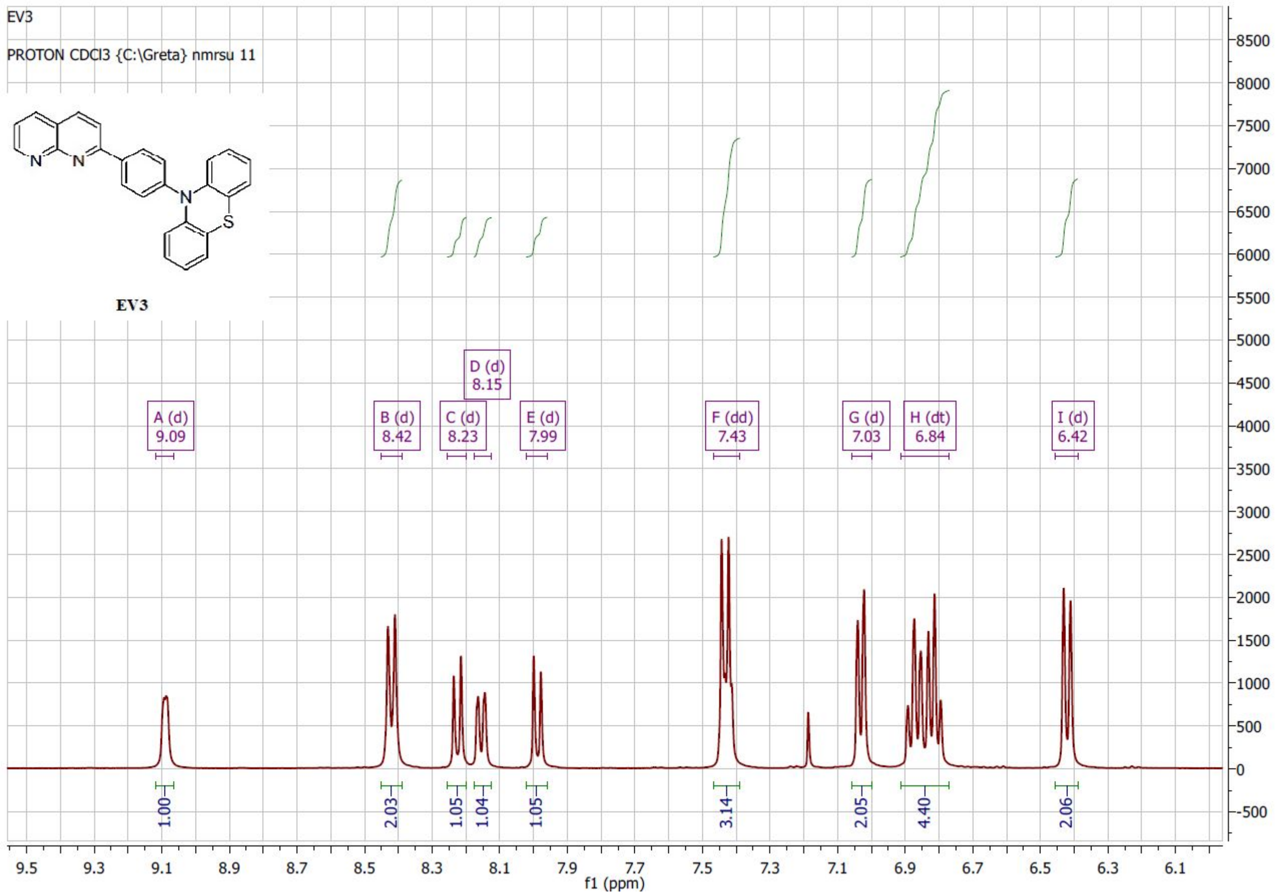
EV3
PROTON CDCl3 {C:\Greta} nmrsu 11
N
N
N
S
EV3
A (d) 9.09
B (d) 8.42
C (d) 8.23
D (d) 8.15
E (d) 7.99
F (dd) 7.43
G (d) 7.03
H (dt) 6.84
I (d) 6.42
1.00
2.03
1.05
1.04
1.05
3.14
2.05
4.40
2.06
f1 (ppm)

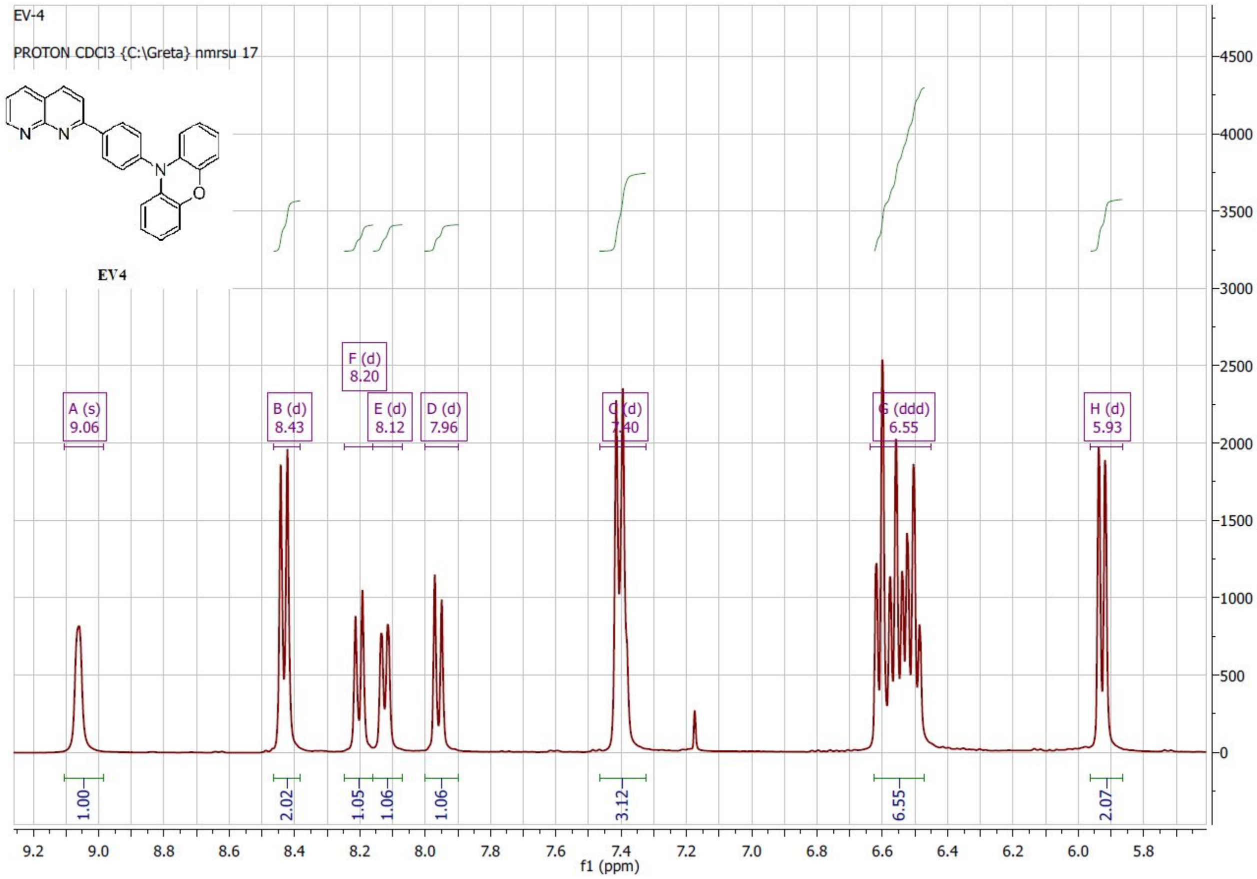

Figure S1.^{1}H NMR spectra of naphthyridine-based compounds **EV1–4**

a) b)

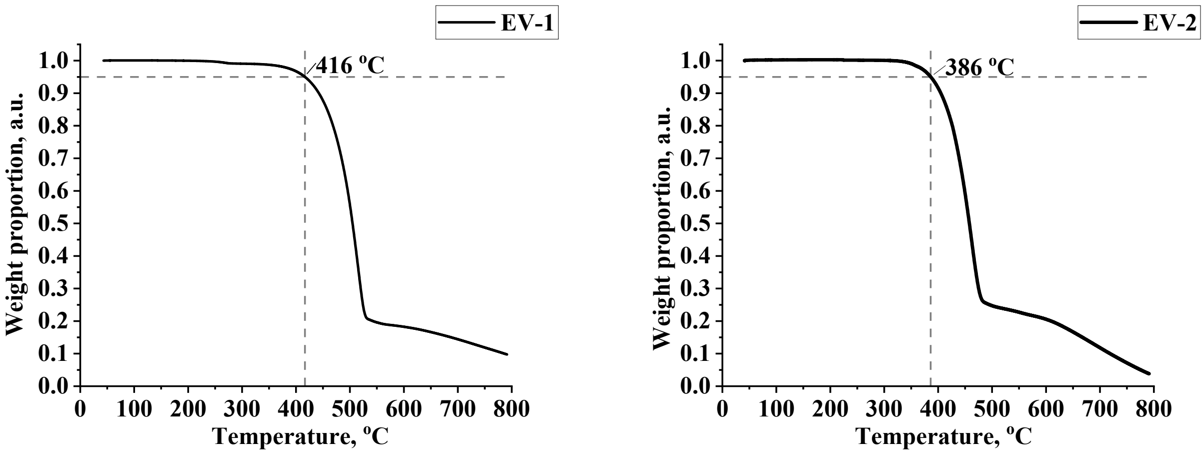

c) d)

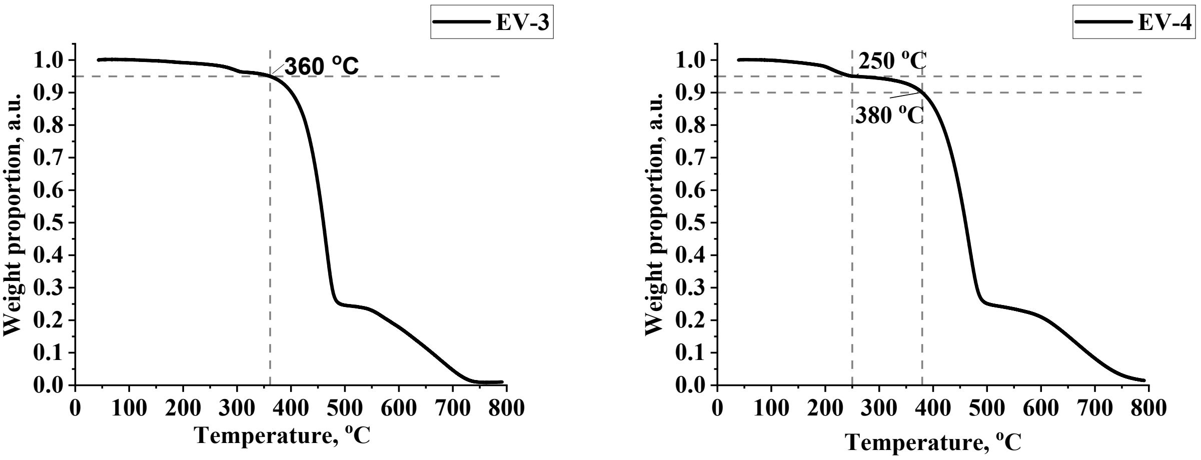

Figure S2. TGA curves of compounds **EV1–4**.

a) b)

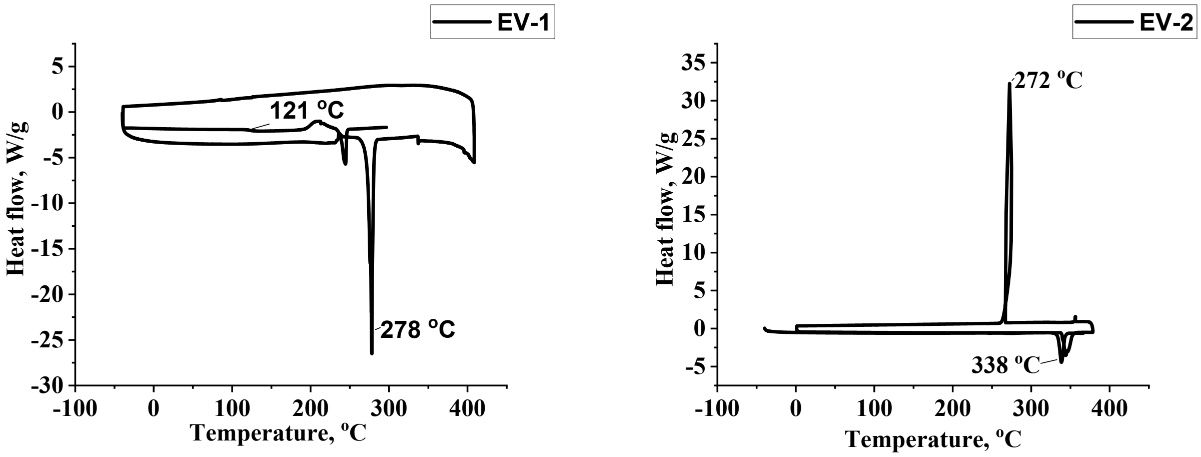

c) d)

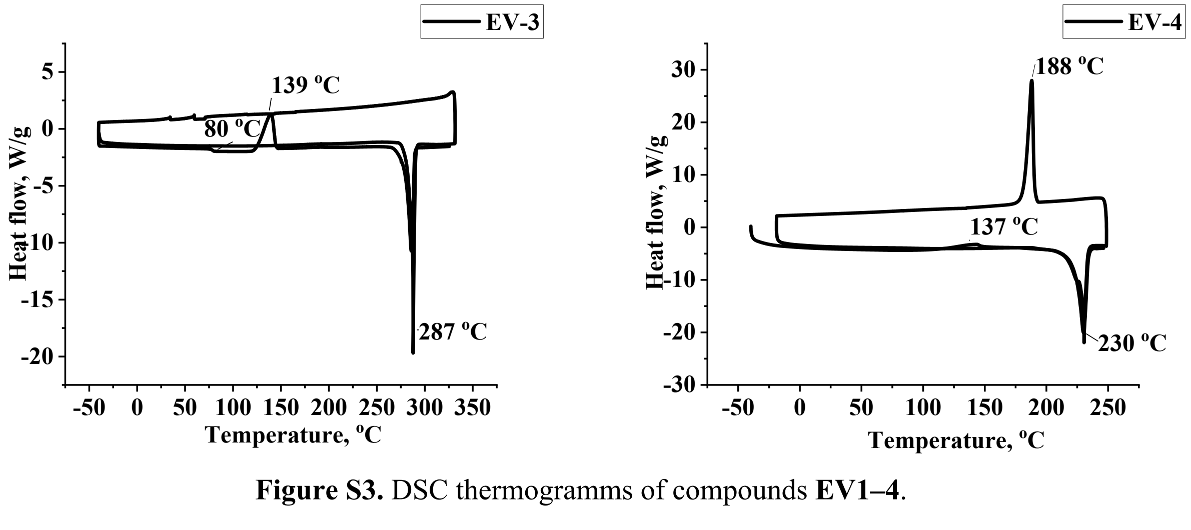

Figure S3. DSC thermogramms of compounds **EV1–4**.

Table S1. Thermal properties of naphthyridine-based compounds **EV1–4**.

**Compound**	**EV1**	**EV2**	**EV3**	**EV4**
**T_m, ℃**	278	338	287	230
**T_g, ℃**	121	-	80	137
**T_{D-5} %, ℃**	416	386	360	380
**T_{Cr}., ℃**	-	272	-	188

T_m, ℃ – melting temperature; T_g, – glass transition temperature; T_{D-5} % – 5% mass loss of sample; T_{Cr} – crystallization temperature.

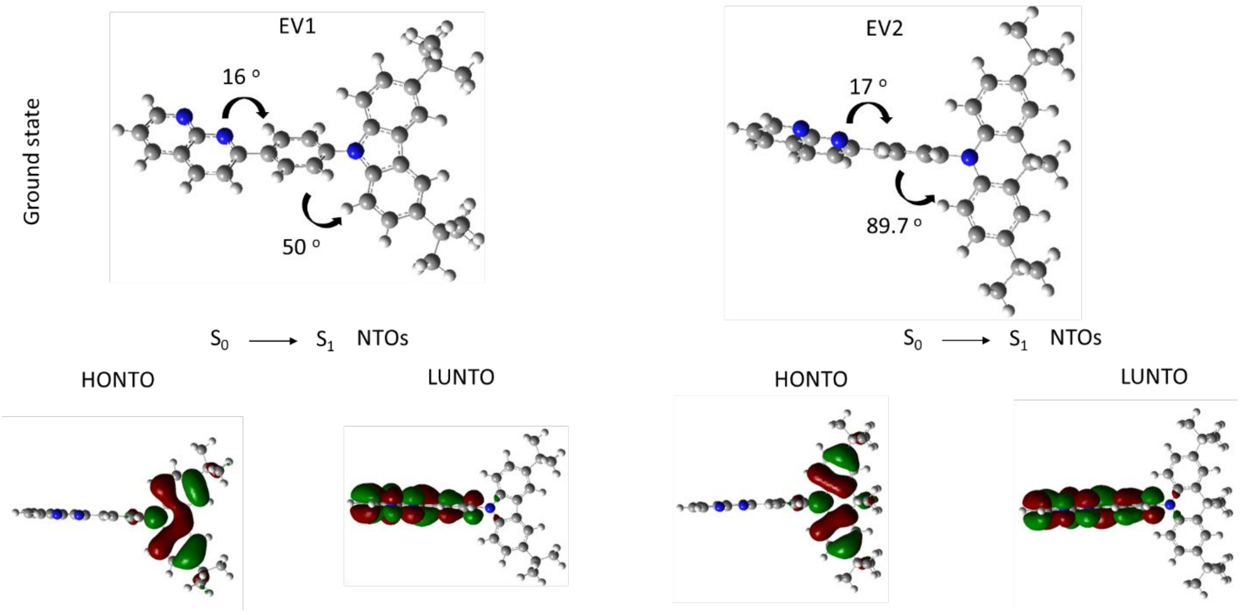

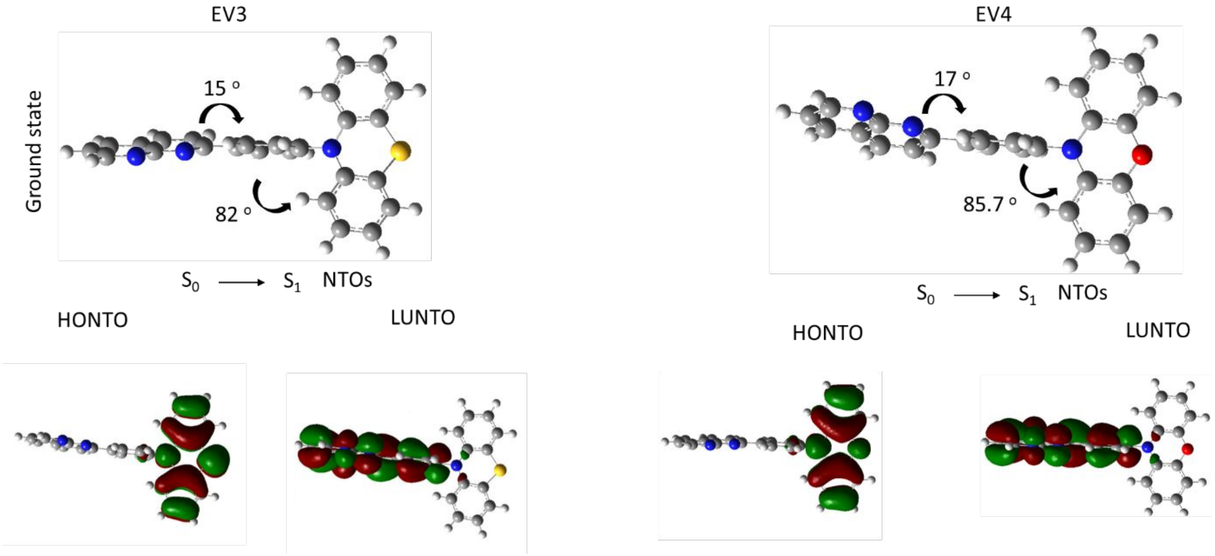

Figure S4. Frontier orbital distributions, optimized geometries and natural transition orbitals of compounds **EV1–4** at the ground and excited states, with the respective energy levels, calculated at B3LYP/6-31G (d,p) level.

a) b)

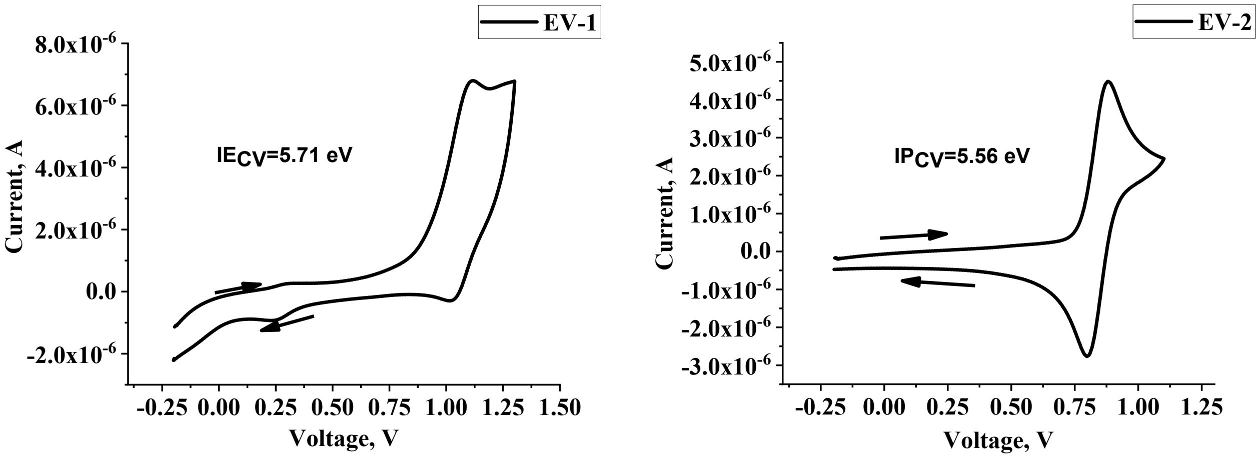

c) d)

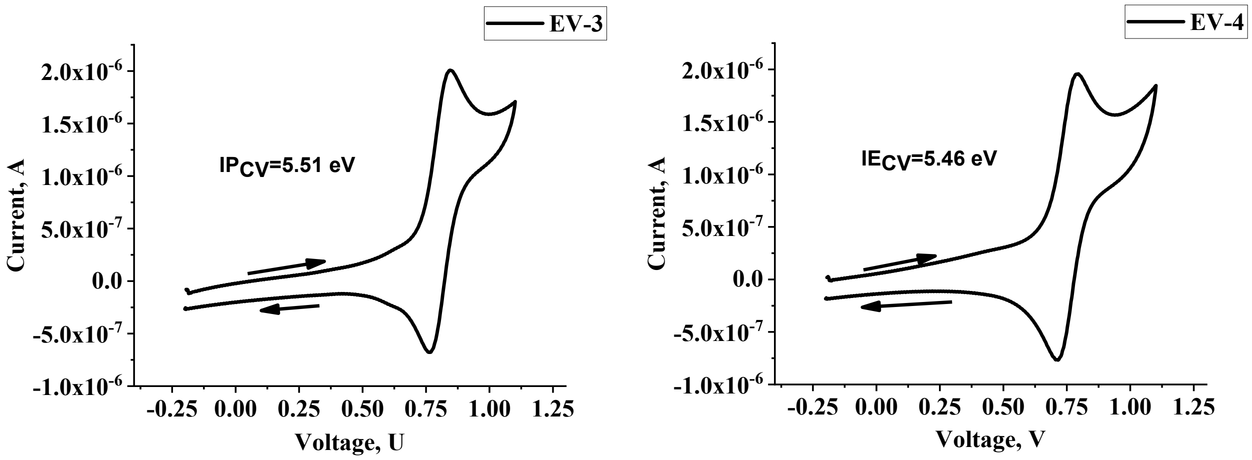

Figure S5. CV voltammograms of compounds **EV1–4**.

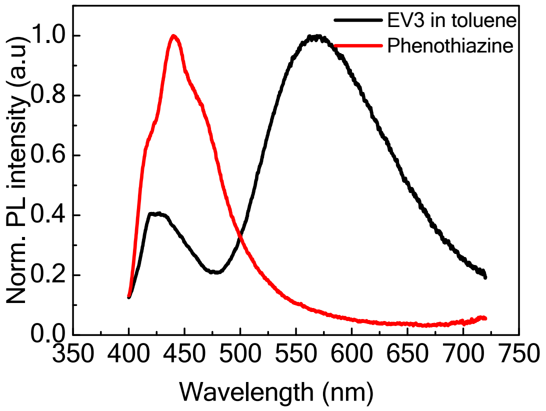

Figure S6. PL spectra of **EV3** and phenothiazine toluene solutions.

a) b) c)

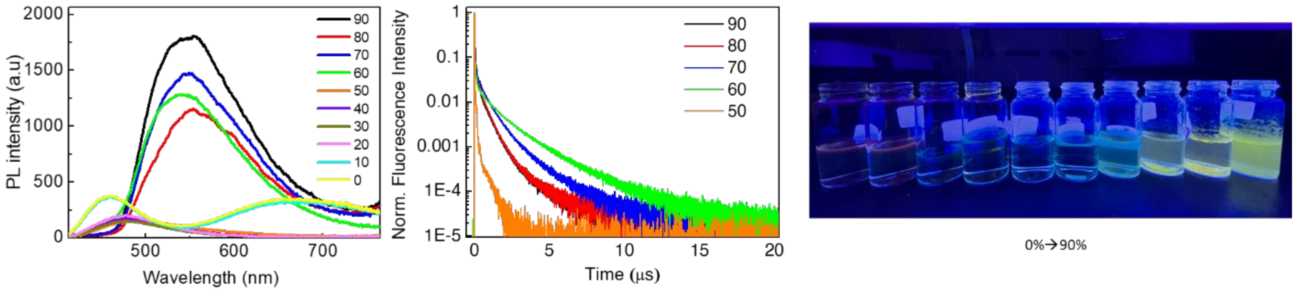

Figure S7. (a) PL spectra, (b) TrPL signals and (c) picture of solution under UV light radiation of **EV3** in THF/water mixtures with different water fractions (fw).

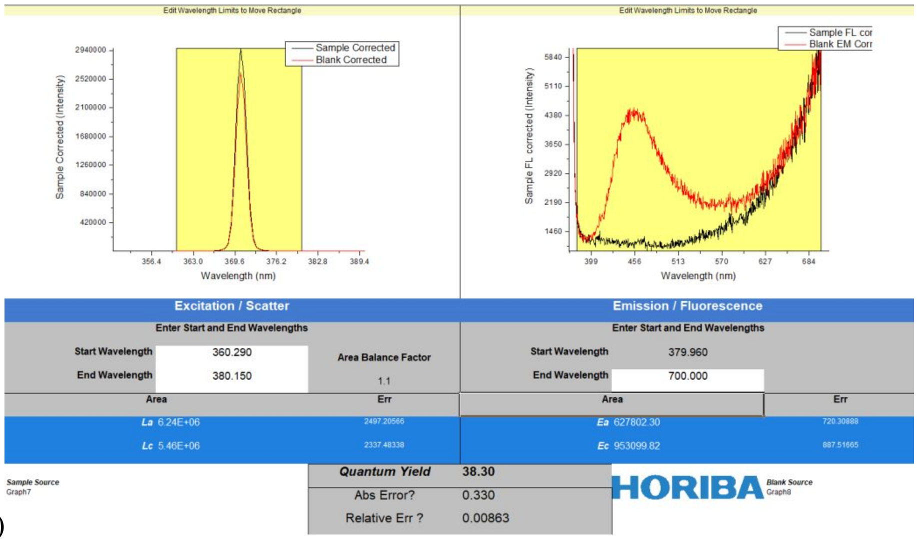
Sample Corrected
Blank Corrected
Sample Corrected (Intensity)
Wavelength (nm)
Sample FL cor
Blank EM Corr
Sample FL corrected (Intensity)
Wavelength (nm)
Excitation / Scatter
Emission / Fluorescence
Enter Start and End Wavelengths
Start Wavelength 360.290
End Wavelength 380.150
Area Balance Factor 1.1
Start Wavelength 379.960
End Wavelength 700.000
Area
Err
La 6.24E+06 2497.20566
Lc 5.46E+06 2337.48338
Ea 627802.30 720.30888
Ec 953099.82 887.51665
Quantum Yield 38.30
Abs Error? 0.330
Relative Err ? 0.00863
Sample Source Graph7
Blank Source Graph8
HORIBA

a)

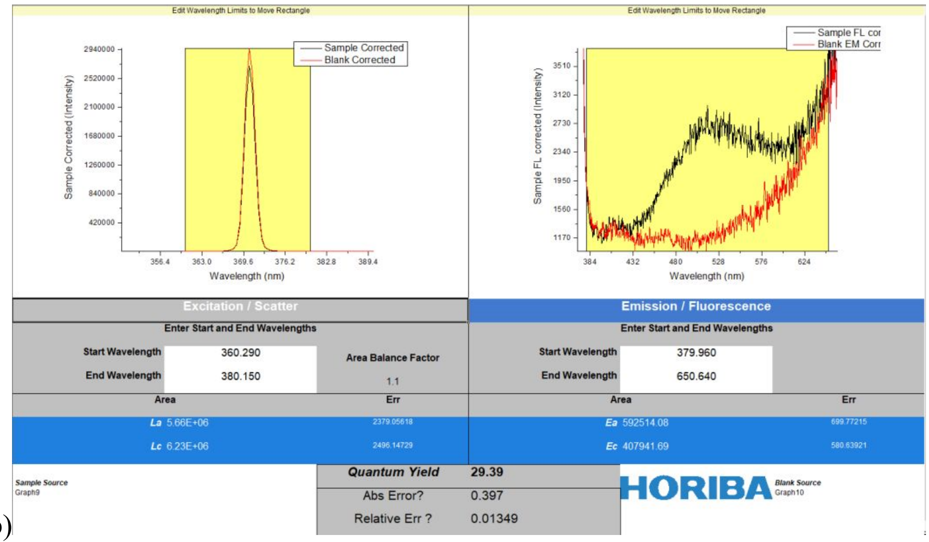
Sample Corrected
Blank Corrected
Sample Corrected (Intensity)
Wavelength (nm)
Sample FL cor
Blank EM Corr
Sample FL corrected (Intensity)
Wavelength (nm)
Excitation / Scatter
Emission / Fluorescence
Enter Start and End Wavelengths
Start Wavelength 360.290
End Wavelength 380.150
Area Balance Factor 1.1
Start Wavelength 379.960
End Wavelength 650.640
Area
Err
La 5.66E+06 2379.05618
Lc 6.23E+06 2496.14729
Ea 592514.08 699.77215
Ec 407941.69 580.63921
Quantum Yield 29.39
Abs Error? 0.397
Relative Err ? 0.01349
Sample Source Graph9
Blank Source Graph10
HORIBA

b)

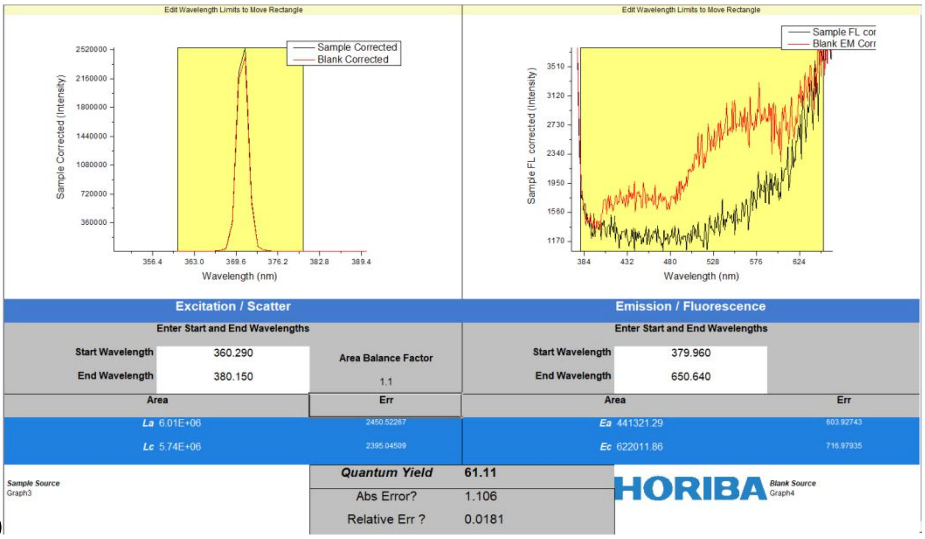
Sample Corrected
Blank Corrected
Sample Corrected (Intensity)
Wavelength (nm)
Sample FL cor
Blank EM Corr
Sample FL corrected (Intensity)
Wavelength (nm)
Excitation / Scatter
Emission / Fluorescence
Enter Start and End Wavelengths
Start Wavelength 360.290
End Wavelength 380.150
Area Balance Factor 1.1
Start Wavelength 379.960
End Wavelength 650.640
Area
Err
La 6.01E+06 2450.52267
Lc 5.74E+06 2395.04509
Ea 441321.29 603.92743
Ec 622011.86 716.97935
Quantum Yield 61.11
Abs Error? 1.106
Relative Err ? 0.0181
Sample Source Graph3
Blank Source Graph4
HORIBA

c)

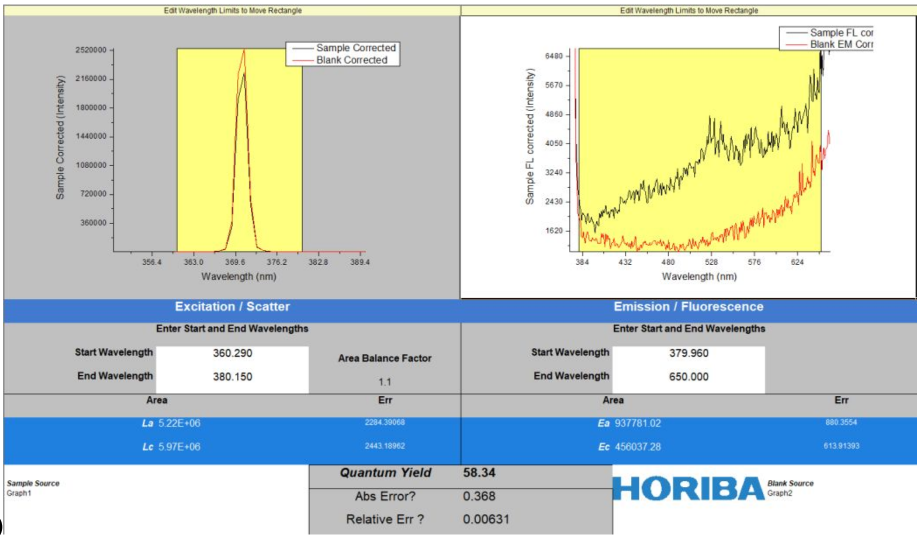

d)

Figure S8. PLQY of thin films of **EV1–4** (10%) doped in *o*-DiCbzBz.

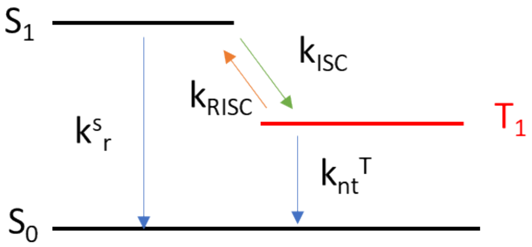

Figure S9. Optical diagram of **EV1–4** (10%) doped in *o*-DiCbzBz. ($k^S r$: radiative decay rate from S_1 to S_0, k_{ISC}: ISC rate from S_1 to T_1, k_{RISC}: reverse ISC rate from T_1 to S_1, k_{nt}^T: nonradiative decay rate from T_1 to S_0)

$k_r^S = \Phi_F k_p \quad k_p = \frac{1}{\tau_p}$, τ_p: prompt flourescence emission lifetime，Φ_F: PLQY of prompt component.

$$k_{ISC} = (1 - \Phi_F) k_p$$

$(1 - \Phi_F)\Phi_{RISC} = \Phi_{TADF}$, Φ_{TADF}: PLQY of delayed component.

$k_{RISC} = \frac{k_p k_d \Phi_{TADF}}{k_{ISC} \quad \Phi_F} \quad k_d = \frac{1}{\tau_d}$, τ_p: delayed flourescence emission lifetime

$$k_{nt}^T = k_d - \Phi_F k_{RISC}$$

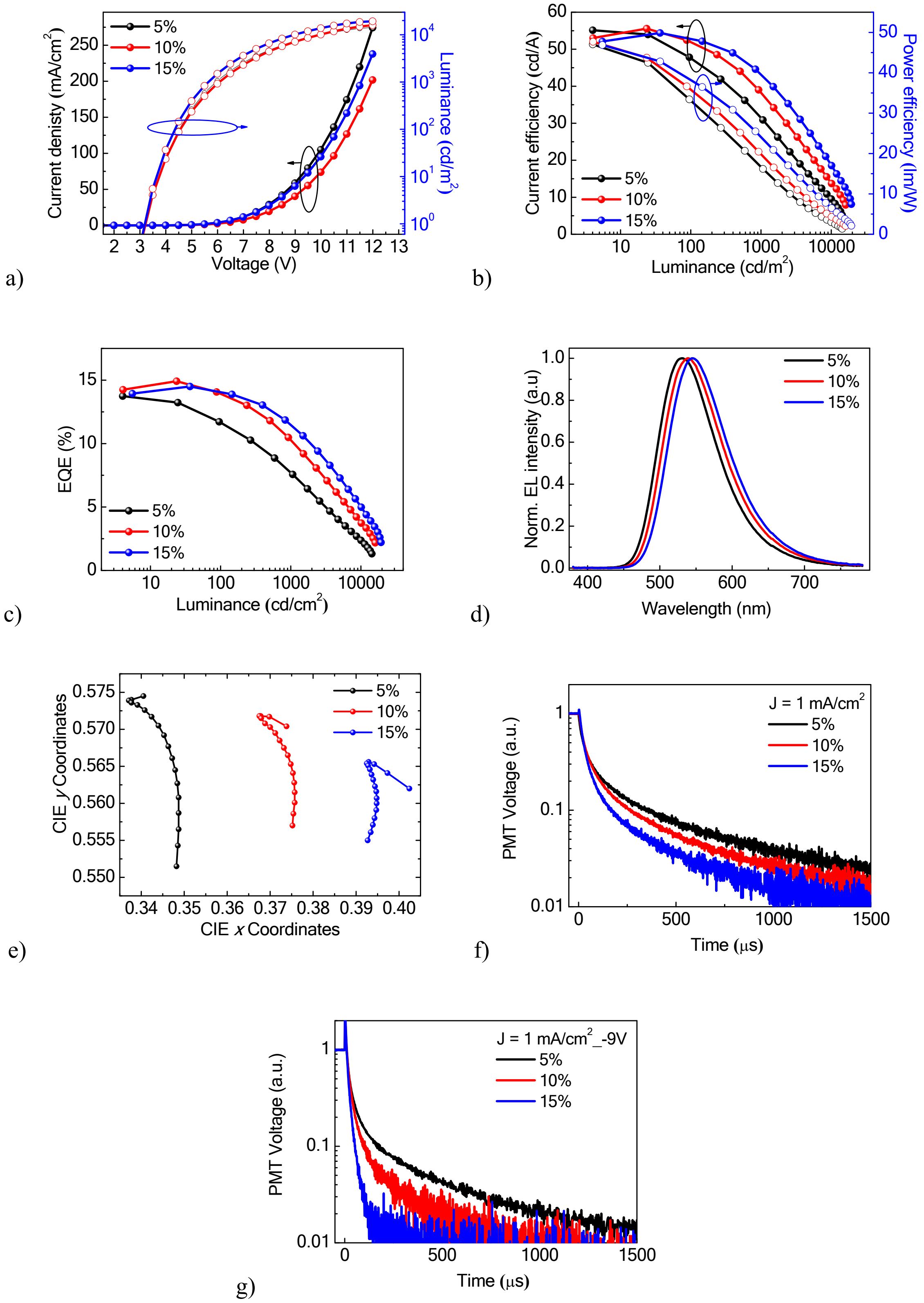

Figure S10. (a) J−V−L, (b) CE and PE−L, (c) EQE−L plots, (d) normalized EL spectra recorded at 5 V, (e) CIE coordinates, TrEL recorded at constant current J = 1 mA/cm^2 (f) without and (g) with -9 V reversed bias at 2000 µs window.

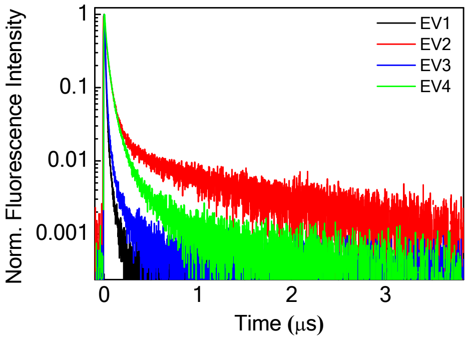

Figure S11. TrPL signals of **EV1–4** in neat film in 4 μs window.

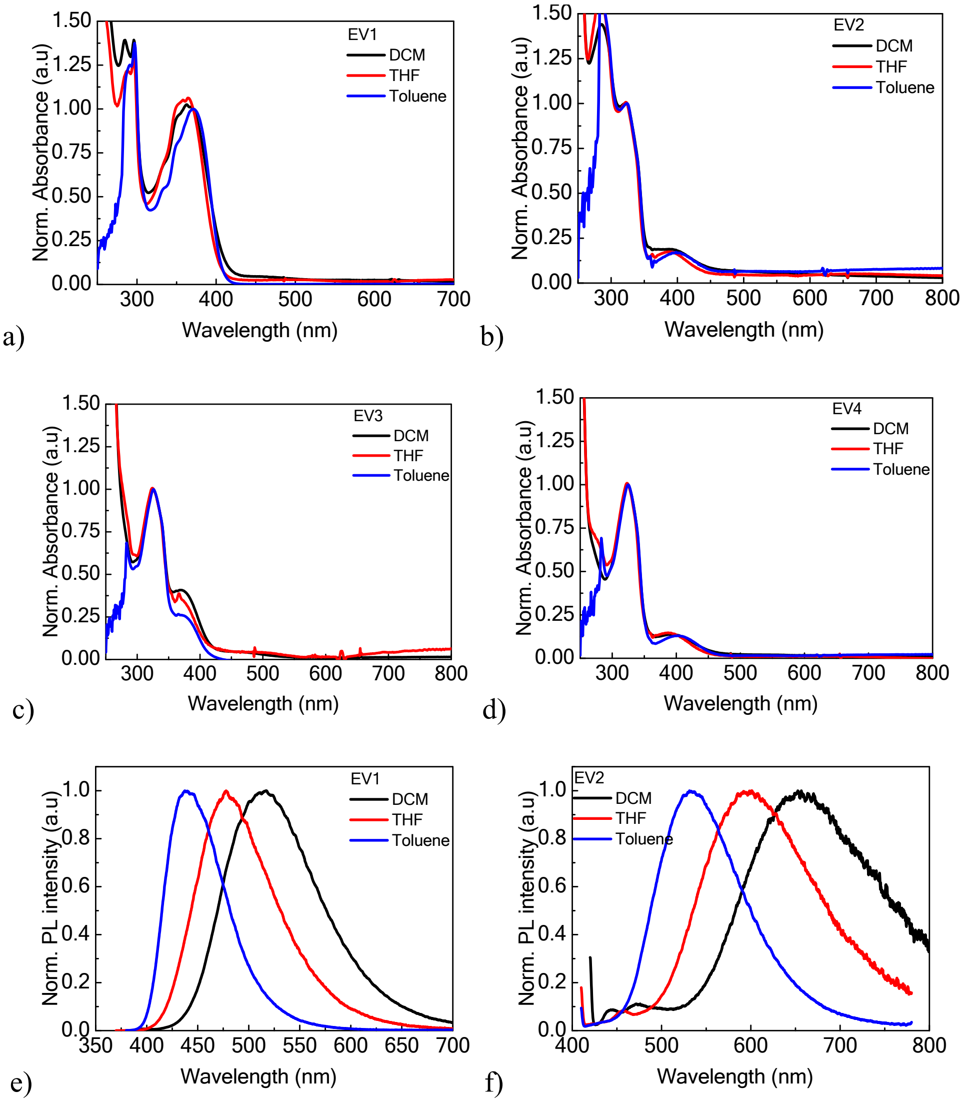

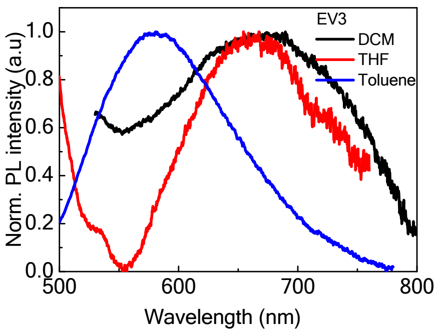

g)

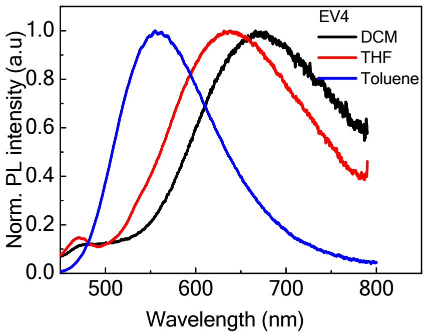

h)

Figure S12. (a)−(d) Normalized absorbance and (e)−(h) normalized PL spectra of EV1−EV4 dissolved in DCM, THF and toluene.

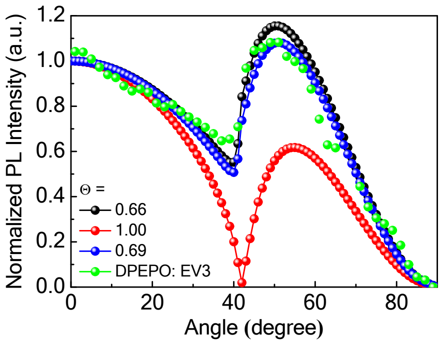

Figure S13. ADPL intensity and simulated orientation order parameter (Θ_{ADPL}) of the molecular mixture of DPEPO host and **EV3** emitter.

References

(1) Gritzner, G.; Kuta, J. Recommendations on Reporting Electrode Potentials in Nonaqueous Solvents (Recommendations 1983). *Pure and Applied Chemistry* **1984**, *56* (4), 461–466. https://doi.org/10.1351/pac198456040461.

(2) Cardona, C. M.; Li, W.; Kaifer, A. E.; Stockdale, D.; Bazan, G. C. Electrochemical Considerations for Determining Absolute Frontier Orbital Energy Levels of Conjugated Polymers for Solar Cell Applications. *Advanced Materials* **2011**, *23* (20), 2367–2371. https://doi.org/10.1002/adma.201004554.

(3) Rybakiewicz, R.; Gawrys, P.; Tsikritzis, D.; Emmanouil, K.; Kennou, S.; Zagorska, M.; Pron, A. Electronic Properties of Semiconducting Naphthalene Bisimide Derivatives—Ultraviolet Photoelectron Spectroscopy versus Electrochemistry. *Electrochimica Acta* **2013**, *96*, 13–17. https://doi.org/10.1016/J.ELECTACTA.2013.02.041.

(4) Becke, A. D. Density-Functional Exchange-Energy Approximation with Correct Asymptotic Behavior. *Physical Review A* **1988**, *38* (6), 3098–3100. https://doi.org/10.1103/PhysRevA.38.3098.

(5) Frisch, M. J.; Trucks, G. W.; Schlegel, H. B.; Scuseria, G. E.; Robb, M. A.; Cheeseman, J. R.; Scalmani, G.; Barone, V.; Petersson, G. A.; Nakatsuji, H.; et al. Gaussian 09, Revision A.02. *Gaussian, Inc. Wallingford CT* **2016**.

(6) Kormos, A.; Móczár, I.; Sveiczer, A.; Baranyai, P.; Párkányi, L.; Tóth, K.; Huszthy, P. Synthesis and Anion Recognition Studies of Novel 5,5-Dioxidophenothiazine-1,9-Diamides. *Tetrahedron* **2012**, *68* (35), 7063–7069. https://doi.org/10.1016/J.TET.2012.06.070.
